# Edge disturbance shapes liana diversity and abundance but not liana‐tree interaction network patterns in moist semi‐deciduous forests, Ghana

**DOI:** 10.1002/ece3.8585

**Published:** 2022-02-20

**Authors:** Bismark Ofosu‐Bamfo, Patrick Addo‐Fordjour, Ebenezer J.D. Belford

**Affiliations:** ^1^ Department of Basic and Applied Biology School of Sciences University of Energy and Natural Resources Sunyani Ghana; ^2^ 98763 Department of Theoretical and Applied Biology Faculty of Biosciences College of Science Kwame Nkrumah University of Science and Technology Kumasi Ghana

**Keywords:** co‐occurrence patterns, ecological networks, edge influence, liana diversity and abundance, modularity, nestedness, specialisation

## Abstract

Edge disturbance can drive liana community changes and alter liana‐tree interaction networks, with ramifications for forest functioning. Understanding edge effects on liana community structure and liana‐tree interactions is therefore essential for forest management and conservation. We evaluated the response patterns of liana community structure and liana‐tree interaction structure to forest edge in two moist semi‐deciduous forests in Ghana (Asenanyo and Suhuma Forest Reserves: AFR and SFR, respectively). Liana community structure and liana‐tree interactions were assessed in 24 50 × 50 m randomly located plots in three forest sites (edge, interior and deep‐interior) established at 0–50 m, 200 m and 400 m from edge. Edge effects positively and negatively influenced liana diversity in forest edges of AFR and SFR, respectively. There was a positive influence of edge disturbance on liana abundance in both forests. We observed anti‐nested structure in all the liana‐tree networks in AFR, while no nestedness was observed in the networks in SFR. The networks in both forests were less connected, and thus more modular and specialised than their null models. Many liana and tree species were specialised, with specialisation tending to be symmetrical. The plant species played different roles in relation to modularity. Most of the species acted as peripherals (specialists), with only a few species having structural importance to the networks. The latter species group consisted of connectors (generalists) and hubs (highly connected generalists). Some of the species showed consistency in their roles across the sites, while the roles of other species changed. Generally, liana species co‐occurred randomly on tree species in all the forest sites, except edge site in AFR where lianas showed positive co‐occurrence. Our findings deepen our understanding of the response of liana communities and liana‐tree interactions to forest edge disturbance, which are useful for managing forest edge.

## Graphical abstract

Edge effects positively and negatively influenced liana diversity in the two forests, and also enhanced liana abundance. Liana‐tree networks were non‐nested, less connected and more specialised and modular, irrespective of edge disturbance. Edge disturbance did not shift the topological roles of most of the lianas, but a few species had their roles changed.

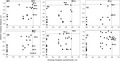

## 1 INTRODUCTION

Lianas are woody climbing plants that are rooted in the soil, but use trees or shrubs to climb to the canopy. Lianas are common in tropical forests, but they are generally more abundant and diverse in disturbed areas such as forest edges and canopy gaps (Zhu & Cao, 2010). Nonetheless, some authors found that lianas were less abundant in some disturbed forests (Addo‐Fordjour et al., 2012). Irrespective of the trend, liana community structure often changes with disturbance in forest ecosystems (Bongers et al., 2020). Human disturbance of forests results in fragmentation (Harper et al., 2005), causing changes in forest structure and microclimatic conditions at edges (Magnago et al., 2015). Such edge‐induced changes tend to be favourable to disturbance‐adapted, light‐demanding species such as lianas (see Hawthorne, 1996; Laurance et al., 2001), but generally disadvantageous to trees (Laurance et al., 2006). The disturbance‐adapted nature of lianas makes them an ideal group of plants which can be used to test edge effects. Previous studies reported that edge effects enhanced liana diversity and abundance in some forests (Addo‐Fordjour et al., 2021; Campbell et al., 2018; Laurance et al., 2001; Ofosu‐Bamfo et al., 2019), but others did not detect changes in liana diversity in response to edge (Mohandass et al., 2014; Ofosu‐Bamfo et al., 2019). Several properties of forest edge such as edge size, edge type, and surrounding matrix type can mediate edge effects on plant community structure (Martino, 2015), and be responsible for the varied responses of community structure to edges in different forests. For example, liana and tree communities may show different responses to forest edge (Addo‐Fordjour & Owusu‐Boadi, 2016). Such edge‐induced changes can alter liana‐tree interactions, as reported for plant‐animal networks (Fagan et al., 1999; Porensky, 2011). Nonetheless, there is scarcity of information on the response of liana‐tree interaction network patterns to forest edge. Studying edge effects on liana community structure and liana‐tree interactions can reveal interesting and unique findings that can contribute towards the development of edge theory.

Species interact to form complex networks of biological communities (Hagen et al., 2012). The species interactions that occur within networks tend to shape ecological communities and drive evolution (Fontaine et al., 2011; Jacobsen et al., 2018). Ecological network approach has been used to study species interactions in more detail, revealing much more information on community structure (Watts et al., 2016). The use of ecological networks thus improves our knowledge on community ecology and the evolutionary processes shaping biological communities (Losapio et al., 2019). Thus, understanding of network patterns would make it possible to predict the ecological and evolutionary consequences of networks. For instance, networks exhibiting modular structure are expected to show a higher stability and robustness, as such a structure would limit diffusion of perturbations through the networks (Thébault & Fontaine, 2010). Médoc et al. (2017) reported that nestedness increases the stability of networks. Moreover, Thébault and Fontaine (2010) revealed that nestedness increases the stability of mutualistic networks, but destabilises antagonistic networks. With regards to evolution, nestedness increases the variation of individual fitness, resulting in a core of species that drive the evolution of the whole community (Bascompte et al., 2003; Cantor et al., 2017; Gómez et al., 2011). Similarly, modularity may also enhance evolution by allowing certain modules to evolve independently of other organisms (see Hansen, 2003).

In spite of the usefulness of ecological network approach as outlined above, it is scarcely used in liana studies, resulting in limited knowledge on liana‐tree interaction networks, and lack of consensus regarding the interaction patterns. Previous studies used different network metrics to characterise liana‐tree interactions. For example, nestedness, a network pattern in which the interactions of less connected species form proper subsets of the interactions of more connected species (Bascompte et al., 2003; Landi et al., 2018; Ponisio et al., 2019), has been used to characterise the structure of liana‐tree networks. Different patterns of nestedness are reported in literature for liana‐tree networks including nested (Sfair et al., 2010) and non‐nested (Addo‐Fordjour & Afram, 2021; Addo‐Fordjour et al., 2016, 2021; Blick & Burns, 2009; Magrach et al., 2016; Ofosu‐Bamfo et al., 2019) structures. Among the studies that did not find nested structure in liana‐tree networks, some reported anti‐nested structure which depicts non‐random assembly (Addo‐Fordjour & Afram, 2021; Addo‐Fordjour et al., 2021; Blick & Burns, 2009; Magrach et al., 2016), while others observed non‐significant nestedness that shows random assembly (Addo‐Fordjour et al., 2016; Ofosu‐Bamfo et al., 2019). Ecological networks can also be compartmentalised into modules whose members interact more among themselves (Carstensen et al., 2016). This phenomenon referred to as modularity, is predicted to stabilise ecological networks (Massol et al., 2017; Thébault & Fontaine, 2010). Species within modular networks perform distinct topological roles, with implications for forest management and conservation (Olesen et al., 2007). Sfair et al. (2010) did not find modular structure in their networks, but Addo‐Fordjour and Afram (2021) recorded significant modular structure in liana‐tree networks.

Specialisation at the network and species levels can cause non‐nested and modular organisation of species (Addo‐Fordjour & Afram, 2021; Castledine et al., 2020; Médoc et al., 2017). Thus, in liana‐tree networks in which coevolution leads to specialisation (Sfair et al., 2015), the networks may tend to be non‐nested and/or modular. Another important metric used to characterise network structure is species co‐occurrence, which describes the frequency of pairs of liana species to co‐occur on the same phorophyte species (Zulqarnain et al., 2016). Species co‐occurrence patterns are useful in inferring the ecological and evolutionary history of liana species, as closely related species tend to have similar niches that increase their chances of co‐occurrence (Zulqarnain et al., 2016). Like the above‐mentioned network metrics, mixed patterns of liana species co‐occurrence have been reported in literature, which include positive co‐occurrence (Addo‐Fordjour et al., 2016; Zulqarnain et al., 2016), negative co‐occurrence (Blick & Burns, 2009, 2011), and random co‐occurrence (Addo‐Fordjour et al., 2016). With the mixed findings on the structure of liana‐tree interactions in literature, there is the need for more studies to be conducted to determine the most consistent patterns. Knowledge of co‐occurrence patterns is important for increasing our understanding of species interactions and predicting community stability and maintenance, and ecosystem functioning, all of which are useful in forest conservation (Vizentin‐Bugoni et al., 2016).

This study determined the response patterns of liana community assemblages and structure of liana‐tree interaction networks to edge in two moist semi‐deciduous forests in Ghana. The forest edges we studied were surrounded by large matrices of crop farmlands, thus making the edges much exposed. The nature and size of land matrix bordering forest edges play a key role in determining the intensity of edge effects on plant community structure (Aragón et al., 2015). To this end, edges bordered by wide land matrices are expected to exert stronger effects on plant communities than edges surrounded by narrow area of land (Addo‐Fordjour & Owusu‐Boadi, 2016). In reality, the existence of a marked contrast in the physiognomy and structure between a forest edge and its surrounding land matrix causes variation in the microclimatic conditions of that forest edge and the interior site (Aragόn et al., 2015). Based on the above, we expected edge effects on liana assemblages and liana‐tree interaction patterns in the two moist semi‐deciduous forests. Edge disturbance permits greater penetration of sunlight into forest edges, and also increases forest edge dryness (Thier & Wesenberg, 2016), both of which can favour liana proliferation. On the basis of the above, we tested the following hypotheses:

1. Liana diversity and abundance would be higher in edge site than non‐edge sites.
2. We expected that as edge disturbance enhances liana abundance at the forest edge, network connectance will increase, resulting in less specialised, nested and non‐modular network structures in edge site, while the networks in the non‐edge sites will be less connected, more specialised, non‐nested and modular.
3. Edge effects will cause shifts in topological roles of liana and tree species due to changes in the distribution and abundance of the species.
4. As sunlight and dry conditions are elevated at edge sites relative to the non‐edge sites, competition of lianas for the resources in edge site may be lower. Moreover, as edge effects tend to cause tree mortality at forest edges (Murcia, 1995), the number of available host species may reduce, increasing liana infestation per host. Thus, we expected that liana species in edge sites would show positive co‐occurrence on host trees, while the species in non‐edge sites will randomly co‐occur on their hosts.

The findings of our study would be useful in the management of forest edges and conservation of edge species. Our study seeks to add valuable information to literature, thus helping to obtain general patterns of liana assemblages and structure of liana‐tree interactions in relation to edge effects. These findings can contribute to the development of a theory on edge effects in view of the fact that there is dearth of information on the role of edge disturbance in shaping the patterns of liana‐tree network structure in forests.

## 2 METHODOLOGY

### 2.1 Study areas

We conducted the study in two moist semi‐deciduous tropical forest ecosystems in Ghana, situated about 150 km part: Asenanyo Forest Reserve (AFR) (latitudes 6^o^17’ and 6^o^36’N; longitudes 1^o^50’ and 2^o^16’W) and Suhuma Forest Reserve (SFR) (latitudes 5°56ʹ and 6°11ʹN; longitudes 2°21ʹ and 2°36ʹW). The forest edges studied in the two forests were created by farming activities around 2010. Thus, crop farmlands are the surrounding matrix bordering the edges. Prior to the farming‐induced edges, the two forests had slightly different disturbance histories. AFR was subjected to selective logging in 1995, whilst SFR underwent conventional logging in 1996. The edge site of SFR harboured a lower tree density (185 individuals/ha) than the deep‐interior site (242 individuals/ha). However, tree density in the edge and deep‐interior sites of AFR were comparable (258 and 267 individuals/ha, respectively). Furthermore, canopy in the SFR was mainly open, whereas that of the AFR was closed to a larger extent. The presence of closed and open canopy in AFR and SFR, respectively, suggest that climatic differences between edge and interior sites of AFR could be more pronounced than those in SFR. Based on the above differences in the structure of the two forests, we expected the patterns of edge effects on liana communities in the forests to differ.

### 2.2 Asenanyo forest reserve

Asenanyo forest reserve is a production forest that was established in the year 1940 and covers an area of 22,800 ha in the Ashanti Region of Ghana (Wiafe, 2014). It is of the moist semi‐deciduous forest ecosystem, with the dominant tree species being *Celtis mildbraedii*, *Triplochiton scleroxylon* and *Entandrophragma* spp. (Forest Services Division, 2010a; Wiafe, 2014). The forest has a bimodal rainy season from April to October (maximum rainfall: May–June; minimum rainfall: September–October) and a dry season from November to March. Annual rainfall range is 1250–500 mm (Hall & Swaine, 1981). Temperature in the reserve ranges from an average of 30.5°C to 21°C, with a mean annual relative humidity of about 84%. AFR has about 20 admitted farms scattered throughout the reserve, the size of each averaging approximately 5 ha (Forest Services Division, 2010a). The reserve also has one admitted community occupying an area of about 955.70 ha (Forest Services Division, 2010a).

### 2.3 Suhuma forest reserve

SFR is also a production forest of about 36,030 ha located in the Sefwi Wiawso Forest District (Hawthorne & Abu‐Juan, 1995). There are 24 admitted farms in the reserve each averaging 11.5 ha (total 276 ha) and one admitted community covering an area of 389 ha (Forest Services Division, 2010b). The reserve is exposed to active logging. Its canopy is discontinuous due to excessive logging activity but still has emergent trees that may reach heights of about 40 m. The forest lies within the moist semi‐deciduous forest zone in Ghana, and thus its vegetation is dominated by tree species such as *C*. *mildbraeddii*, *Baphia nitida*, *Nesogordonia papaverifera*, *Microdesmis puberula*, *Khaya ivoriensis*, *Daniella ogea* and *Dacryodes klaineana* (Hall & Swaine, 1981). The forest reserve experiences two distinct seasons: the dry season and the rainy season. The rainy season is from April to October, whereas December to March marks the dry season. Average annual rainfall is between 1300 and 1600 mm. Mean annual temperature ranges between 26 and 29°C, and relative humidity is usually above 90% in the rainy season and falls to 60% during the dry season (Forest Services Division, 2010b).

### 2.4 Sampling design and data collection

A total of eight 50 × 50 m plots were randomly established in each of three forest sites, namely, edge, interior and deep‐interior. Each forest site had two randomly demarcated and independent sampling areas, each of which contained four plots. The edge site was defined as 0–50 m from the forest edge, while interior and deep‐interior sites were 200 m and 400 m from the forest edge, respectively. Variable penetration distances of edge have been reported in previous studies. These studies revealed that edges can extend up to 100 m from the forest edge, while other studies also detected edge effects up to 300 m (Flaspohler et al., 2001; Gascon et al., 2000; Laurance et al., 2018; Liu & Taylor, 2002). Thus, we set our two interior sites 100 m beyond each of the aforementioned edge penetration distance limit, resulting in 200 m and 400 m distances from the forest edge.

We surveyed and identified all lianas with diameter (at 1.30 m from the rooting base) ≥1 cm as well as trees (diameter at breast height ≥10 cm) that carried lianas in the plots. The minimum inter‐plot distance in the sampling areas was 150 m. Plant species were identified by a plant taxonomist, and through the use of herbarium specimens and identification guides (Hawthorne, 1990; Hawthorne & Jongkind, 2006).

### 2.5 Data analysis

#### 2.5.1 Community structure

We used species richness, Shannon diversity index and species evenness to characterise liana diversity in the forest sites. A rarefaction‐extrapolation technique was used to standardise species richness based on a constant number of individuals using iNEXT package in R. We computed Shannon diversity index and species evenness with PAST statistical package version 2.17c (Hammer et al., 2001) and tested the significance of the differences in the indices among the forest sites using permutation tests in the PAST software. Computation of Shannon diversity index (H′) and species evenness index (*E*) was based on the following equations:

$$H\prime =‐\sum_{i=1}^{s}pi\ln\;pi\;\;\;\text{and}\;\;\;E=\left(\exp\;H\prime \right)/\left(S\right)$$

where, *pi* = proportion of the *i*th species, and In*pi* = natural log of *pi*, *S* = species richness

Community abundance of lianas was compared among the forest sites by running nested ANOVA, where sampling area was nested within forest site. We employed aov function in the stats package in R to perform the nested ANOVA.

Using the equation of Harper et al. (2005, 2015), we calculated magnitude of edge influence (MEI) on abundance for individual liana species with abundance ≥10 stems. The equation is given as: $\text{MEI}=\frac{e‐i}{e+i}$, where *e* = species abundance in edge site, and *i* = species abundance in non‐edge site, which was obtained by finding the average of the values of interior and deep‐interior sites. The values of MEI ranges from −1 (negative edge influence) to +1 (positive edge influence). MEI value of zero indicates no edge influence. The strength of MEI was determined as follows (Ofosu‐Bamfo et al., 2019): 0 (no edge influence), ≤0.19 (very weak), 0.20–0.39 (weak), 0.40–0.59 (moderate), 0.60–0.79 (strong), 0.80–1.0 (very strong).

#### 2.5.2 Network structure of liana‐tree interactions

Liana‐tree network structure was quantified using the following network metrics: (1) connectance and specialisation asymmetry, (2) degree of specialisation (H2’, d’), (3) nestedness, (4) modularity, (5) module connectivity and interactions (c and z values), (6) species co‐occurrence. We used quantitative liana‐tree species matrices except in the species co‐occurrence test where binary matrices were employed. Each of matrices was made up of liana species assigned to rows and tree species assigned to columns. We also represented the various networks in graphs using plotweb function in the bipartite package in R.

1. Network connectance and specialisation asymmetry

Weighted connectance was calculated to express network connectance in the study. It is defined as the linkage density divided by number of species in the network (van Altena et al., 2016; Dormann, 2021). The values of weighted connectance range from 0 (no connectance) to 1 (perfectly connected). Weighted connectance was run with the networklevel function in the bipartite package.

Similarly, the networklevel function was used to calculate specialisation asymmetry of the networks.

2. Degree of specialisation

The degree of specialisation was determined for the various networks and the individual species in the networks as follows:

Using the H2’ index, we quantified network specialisation of the various forest sites. The index measures the extent to which observed interactions deviate from the interactions that would be expected given the marginal totals of the interactions per species (Blüthgen et al., 2006). Generally, higher values of the H2’ index indicate that the species in the network are more selective, resulting in higher specialisation of the network. The index ranges from 0 (no specialisation) to 1 (complete specialisation). The H2’ index was run with H2fun function in the bipartite package.

The degree of species specialisation was determined by calculating d’ index, using dfun function in the bipartite package. This index is defined as the deviation from a conformity expected by the overall utilisation of potential partners (Blüthgen et al., 2007).

3. Nestedness

Nestedness occurs when the more specialist species interact only with subsets of the species interacting with the more generalist species (Bascompte et al., 2003; Ponisio et al., 2019). This means that generalists interact with one another, and specialists tend to interact with generalists, but specialist–specialist interactions are often absent (Bascompte et al., 2003). We calculated weighted nestedness metric, WNODF with the network‐level function in bipartite package in R (Dormann, 2021), in accordance with the nestedness equation of Almeida‐Neto and Ulrich (2010). The WNODF metric ranges from 0 (fully non‐nested) to 100 (fully nested). There are two forms of non‐nested pattern described in literature: (1) when nestedness value is consistent with the null model expectation, and (2) when nestedness value is significantly less than that of the null model. The aforementioned patterns of nestedness refer to two different community assemblies (random and non‐random assembly, respectively) and therefore must be distinguished. We therefore used anti‐nestedness to refer to the situation where observed nestedness values were significantly lower than those expected by chance, whereas we referred to networks that presented observed nestedness values which were consistent with null model expectation as not nested.

4. Modularity

We measured modularity index (Q) with the DIRTLPAwb+algorithm using computeModules function within the bipartite package (Beckett, 2016). Modularity measures the tendency of a network to form modules of interacting species, which interact more with one another than with species of other modules (Carstensen et al., 2016; Dormann, 2021). The Q index ranges from 0 for networks with clustering not different from random to 1 for networks with perfect modules. The Q index calculation followed the equations in Newman (2006).

5. Test of statistical significance of the metrics

The above mentioned network metrics (i.e. connectance, degree of specialisation, nestedness, modularity) were tested for their statistical significance by generating 1,000 null models and comparing them with the observed metric values using the Patefield algorithm (Patefield, 1981) in the bipartite package.

6. Module connectivity and interactions

The topological roles of liana and tree species with respect to network modularity were assessed based on the number of links of the species. We achieved this by calculating the weighted standardised among‐module connectivity (c) and within‐module interactions (z), using species strength of interaction (Watts et al., 2016). To obtain the corresponding appropriate c and z thresholds for the species, we generated 100 null models of the original networks using DIRTLPAwb + algorithm, and 95% quantiles as thresholds of c and z values. Based on the c and z values generated, the species were grouped into four categories of topological roles (Olesen et al., 2007) indicated below:

- Peripherals: species with lower c and z values compared to the threshold values. They are specialist species with few links, that are mostly or exclusively within their own modules (Watts et al., 2016).
- Connectors: made up of species with higher c values and lower z values compared to the threshold values. Connectors are generalist species that have several links, but the majority of the links occur outside their own modules (Larson et al., 2014; Watts et al., 2016).
- Module hubs: made up of species with higher z values and lower c‐values compared to the threshold values. They are highly connected generalist species that link to many species within their own modules, but with a few species outside the modules (Larson et al., 2014; Watts et al., 2016).
- Network hubs: species with higher c and z values compared to the threshold values. Network hubs are super‐generalist species that have links within their own modules and among other modules (Larson et al., 2014). They are important for their own module and the entire network (Watts et al., 2016).

7. Relationship between species abundance and number of interactions of lianas

Pearson's correlation test was performed between abundance and number of interactions of liana species using cor.test function in stats package in R. The correlation analysis was run to assess the relationship between liana species abundance and their number of interactions (links), as a proxy for the relationship of liana species abundance with their topological roles. The relationships were expressed in scatter plots using ggscatter function in ggpubr package of R. The correlation analysis was run on log‐transformed data.

8. Species co‐occurrence

Liana species co‐occurrence patterns were determined with the cooc_null_model function from EcoSimR package (Gotelli et al., 2015). We used the C‐score metric, which is the average number of checkerboards for two species (Stone & Roberts, 1990), to measure species co‐occurrence. The metric was calculated according to the equation described by Almeida‐Neto and Ulrich (2010). To assess the patterns of co‐occurrence, 10,000 null models were generated by the quasiswap algorithm and compared with the observed c‐score values. The c‐score measures the tendency of species to not co‐occur (Stone & Roberts, 1990). Thus, the greater the c‐score in relation to the null model, the greater the tendency of the species to not co‐occur (i.e. segregation) and the smaller the c‐score value in relation to the null model, the higher the tendency of species to co‐occur (i.e. aggregation).

## 3 RESULTS

### 3.1 Liana community structure

**FIGURE 1:**
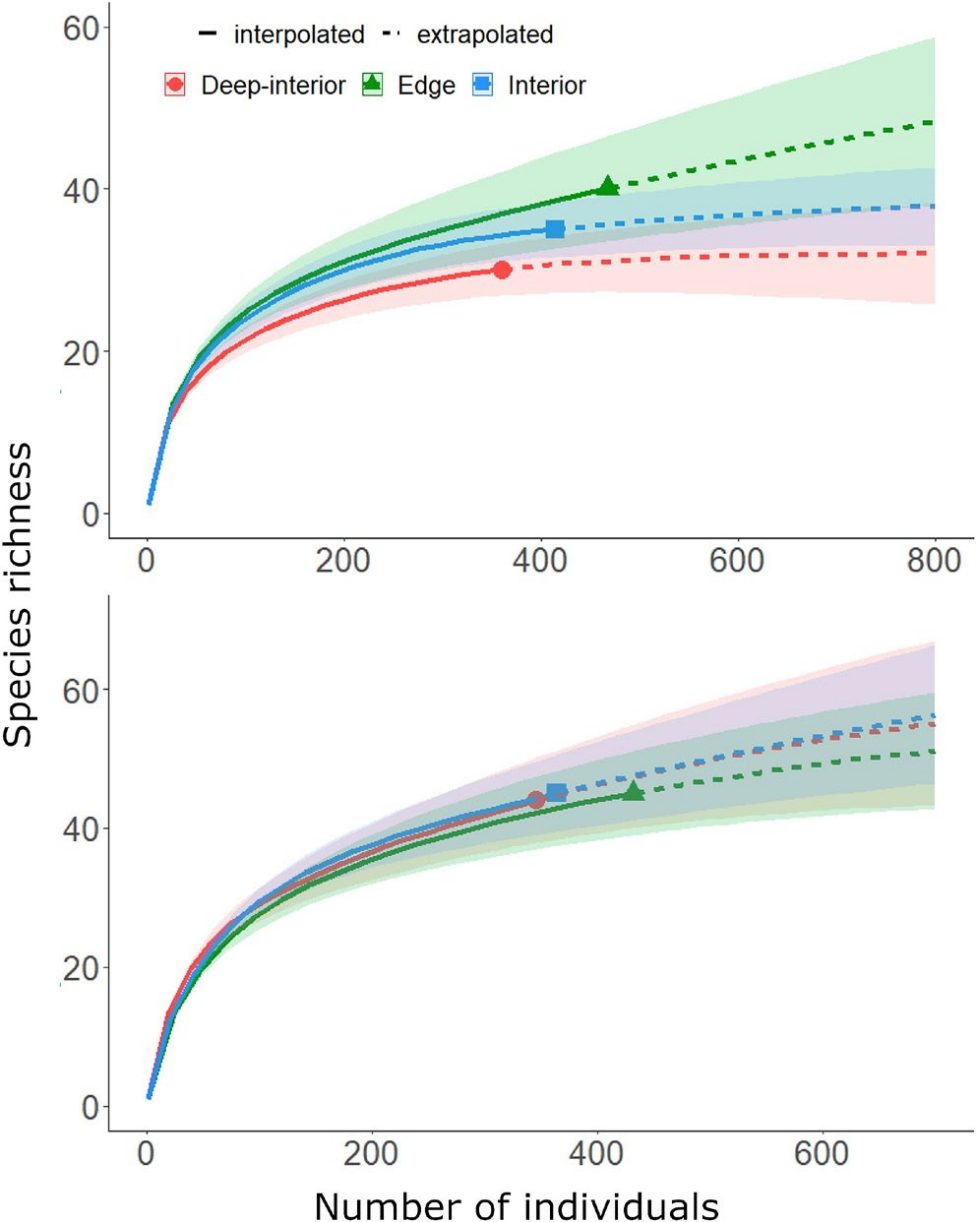
Individual‐based rarefaction‐extrapolation curves showing liana species richness patterns in the three forest sites of the two moist semi‐deciduous forests in Ghana: (a) Asenanyo Forest Reserve, (b) Suhuma Forest Reserve. The solid lines show the rarefaction (interpolation) curves from the reference sample, while the dashed lines indicate the extrapolation curves. The symbols ending the rarefaction curves (see also legend) represent observed number of individuals for the forest sites

There were more liana species in edge site (40 species) than interior site (35 species), which in turn had more species than deep‐interior site (30 species) in AFR (Table 1). Both the rarefaction and extrapolation curves attested to this observation (Figure 1a). The rarefaction curves did not reach asymptote, showing there could be more undetected species in the forest sites. Overall, a total of 49 species were identified in the AFR. The species in edge, interior and deep‐interior sites belonged to 28 genera and 15 families, 26 genera and 16 families, and 24 genera and 12 families, respectively. Edge and interior sites had similar Shannon diversity index (*p* = .506; H’ = 2.94 and 2.89, respectively), while each of them supported significantly higher Shannon diversity index than deep‐interior site (H’ = 2.73) (*p* = .008 and 0.046, respectively). Species evenness (*E*) was similar among all the forest sites in AFR (*p* > .05; edge: *E* = 0.48, interior: *E* = 0.52, deep‐interior: *E* = 0.51).

**TABLE 1:** Liana species abundance and MEI in edge and non‐edge sites in two moist semi‐deciduous forests in Ghana (ES: edge site, IS: interior site, DIS: deep‐interior site, MEI: magnitude of edge influence)

Liana species and families	Asenanyo Forest Reserve				Suhuma Forest Reserve			
	ES	IS	DIS	MEI	ES	IS	DIS	MEI
Apocynaceae								
*Alafia barteri* Oliver	40	33	41	0.04	20	16	22	0.03
*Alafia* sp.	29	13	6	0.51	15	9	9	0.25
*Gongronema latifolium* Benth.	0	0	0		0	1	0	
*Landolphia dulcis* (Sabine ex G. Don) Pichon	1	0	2		1	4	1	
*Landolphia hirsuta* (Hua) Pichon	0	0	0		9	6	9	0.09
*Landolphia owariensis* P. Beauv.	0	0	0		1	1	0	
*Motandra guineensis* (Thonn.) A.DC.	39	41	13	0.18	39	26	18	0.28
*Oncinotis nitida* Benth.	0	0	0		0	0	2	
*Parquetina nigrescens* (Afzel.) Bullock	0	0	0		0	1	0	
*Strophanthus hirsutus* H. Hess	1	0	0		4	0	0	
*Strophanthus hispidus* DC.	1	0	0		0	0	0	
*Strophanthus preussii* Engl. & Pax	8	21	23	−0.52	0	0	0	
*Strophanthus sarmentosus* DC.	0	0	0		6	12	1	−0.01
Celastraceae								
*Hippocratea myriantha* Oliv.	0	0	0		0	0	1	
*Salacia debilis* (G. Don) Walp.	3	0	0		2	5	2	
*Salacia elegans* Welw. ex Oliv.	45	43	32	0.09	15	4	20	0.11
*Salacia lateritia* N. Halle	8	1	0		0	0	0	
*Salacia leptoclada* Tul.	0	0	0		0	3	0	
*Salacia macrantha* A.C. Sm.	0	0	0		0	4	0	
*Salacia preussii* Loes	0	1	0		0	0	0	
*Salacia cerasifera* Welw. Ex Oliv.	0	0	0		0	5	0	
*Salacia staudtiana* Loes. ex Fritsch	0	0	0		0	1	1	
*Salacighia letestuana* (Pellegr.) Blakelock	0	0	0		1	0	0	
*Simirestis staudtii* (Loes.) N. Halle	0	0	0		0	0	2	
Combretaceae								
*Combretum acutum* M.A. Lawson	0	13	0	−1.00	0	1	0	
*Combretum comosum* G. Don	18	3	21	0.20	0	0	3	
*Combretum fuscum* Planch. ex Benth.	0	0	0		0	0	1	
*Combretum micranthum* G. Don	1	0	0		0	0	0	
*Combretum mucronatum* Schumach. & Thonn.	0	0	0		2	4	5	−0.38
*Combretum oyemense* Exell	16	3	8	0.49	0	0	0	
*Combretum paniculatum* Vent.	6	14	0	−0.08	7	9	9	−0.13
*Combretum racemosum* P. Beauv.	0	0	0		9	4	7	0.24
*Combretum sordidum* Exell	0	0	0		2	0	0	
*Combretum* sp.	0	0	0		0	1	0	
*Combretum tarquense* Clark	0	0	0		5	1	16	−0.23
Connaraceae								
*Agelaea obliqua* (P. Beauv.) Baillon	0	2	0		0	0	3	
*Agelaea trifolia* (Lam.) Baill.	1	1	3		1	2	0	
*Castanola paradoxa* (Gilg) Schellenb.	0	0	0		1	0	0	
*Cnestis ferruginea* Vahl ex DC.	0	0	0		0	1	0	
*Connarus africanus* Lam.	0	0	0		0	7	0	
Convolvulaceae								
*Calycobolus africanus* (G. Don) Heine	19	23	7	0.12	25	25	23	0.02
*Calycobolus heudelotii* (Baker ex Oliver) Heine	4	0	0		0	1	1	
*Neuropeltis acuminata* (P. Beauv.) Benth.	8	3	17	−0.11	16	10	18	0.33
*Neuropeltis prevosteoides* Mangenot	0	0	1		4	4	18	−0.12
Dichapetalaceae								
*Dichapetalum dewevrei* De Wild. & T. Durand	0	0	0		1	0	0	
*Dichapetalum pallidum* (Oliver) Engler	0	1	2		1	0	0	
Dilleniaceae								
*Tetracera affinis* Hutch.	2	3	1		0	0	0	
Euphorbiaceae								
*Manniophyton fulvum* Mull. Arg.	0	3	0		17	6	7	0.45
Hernandiaceae								
*Illigera pentaphylla* Welw.	0	0	0		2	0	1	
Icacinaceae								
*Chlamydocarya macrocarpa* A. Chev.exHutch.&D	0	2	0		0	0	0	
Lamiaceae								
*Clerodendrum* sp.	0	0	0		1	0	0	
*Clerodendrum umbellatum* Poir	0	0	0		1	0	0	
Fabaceae								
*Acacia kamerunensis* Gand.	1	0	2		5	7	15	−0.38
*Acacia pentagona* (Schum& Thonn.)Hook f.	10	11	9	0.00	14	25	26	−0.29
*Baphia capparidifolia* Baker	0	0	0		1	0	0	
*Caesalpinia cucullata* Roxb.	16	3	3	0.68	0	1	1	
*Dalbergia hostilis* Bentham	3	3	0		3	1	1	
*Dalbergia oblongifolia* G. Don	0	0	1		0	0	0	
*Dalbergiella welwitschii* (Baker) Baker f.	6	3	5	0.20	3	11	1	−0.33
*Griffonia simplicifolia* (DC.) Baill.	44	37	50	0.01	63	33	25	0.37
*Leptoderris sassandrensis* Jongkind	0	0	0		4	2	0	
*Leptoderris cyclocarpa* Dunn	0	0	0		1	0	0	
*Leptoderris micrantha* Dunn	11	3	14	0.13	2	8	1	−0.38
*Leptoderris miegei* Ake Assi & Mangenot	1	0	0		2	0	8	
*Leucomphalos libericus* Breteler	0	0	0		1	0	0	
*Mezoneuron benthamianum* Baill.	0	0	0		6	0	0	
*Millettia chrysophylla* Dunn	84	74	69	0.08	86	84	34	0.44
*Millettia lucens* (Scott‐Elliot) Dunn	1	0	2		0	0	0	
Linaceae								
*Hugonia planchonii* Hook.f.	0	0	0		0	1	0	
*Hugonia rufipilis* A. Chev. ex Hutch. & Dalziel	0	0	0		1	0	0	
Loganiaceae								
*Strychnos campicola* Gilg	5	8	7	−0.20	0	0	0	
*Strychnos longicaudata* Gilg	0	0	0		9	3	6	0.33
*Strychnos malacoclados* C.H. Wright	0	0	0		0	3	5	
Malpighiaceae								
*Acridocarpus smeathmannii* (DC.) Guill. & Perr.	1	0	0		0	0	0	
*Grewia hookeriana* Exell & Mendonca	1	4	2		0	0	0	
*Grewia malacocarpa* Mast.	0	0	0		7	1	7	0.27
Menispermaceae								
*Tiliacora dielsiana* Hutch. & Dalziel	9	12	12	−0.14	10	5	8	0.21
*Triclisia patens* Oliv.	1	3	1		0	0	1	
Moraceae								
*Ficus* sp.	0	0	0		0	0	2	
Phyllantaceae								
*Phyllanthus* sp.	0	0	0		0	0	1	
Piperaceae								
*Piper guineense* Shumach. & Thonn.	1	0	0		0	4	1	
Polygonaceae								
*Afrobrunnichia erecta* (Asch.) Hutch. & Dalziel	3	2	2		0	0	2	
Rubiaceae								
*Morinda morindoides* (Baker) Milne‐Redh.	10	8	0	0.43	0	0	0	
*Mussaenda tristigmatica* Cummins	1	0	0		0	0	0	
Sapindaceae								
*Paullinia pinnata* Linne	2	15	3	−0.64	0	0	0	
Vitaceae								
*Cissus adenocaulis* Steud. ex A. Rich	6	3	1		6	1	1	
*Cissus silvestris* Tchoume	0	1	0		0	0	0	

Liana species richness was comparable among the three sites in SFR (edge site: 45 species; interior site: 45 species; deep‐interior site: 44 species) (Table 1). The rarefaction and extrapolation curves of the forest sites depicted a similar trend, with the curves showing that there could be more undetected species in the three forest sites. In all, there were 70 liana species identified within SFR. The species belonged to 29 genera and 15 families in edge site, 27 genera and 13 families in interior site, and 27 genera and 16 family in deep‐interior site. (Figure 1b). Edge and interior sites had similar Shannon diversity index (*p* = .530; H’ = 2.99 and 3.05, respectively), while each of them supported significantly lower Shannon diversity index than deep‐interior site (H’ = 3.25) (*p* = .004 and .026, respectively). Species evenness (*E*) was similar among edge and interior sites in SFR (*p* = .686; edge: *E* = 0.45, interior: *E* = 0.47). Liana species in deep‐interior site had a significantly higher evenness (*E* = 0.58) than evenness in edge (*p* = .002) and interior (*p* = .004) sites.

**FIGURE 2:**
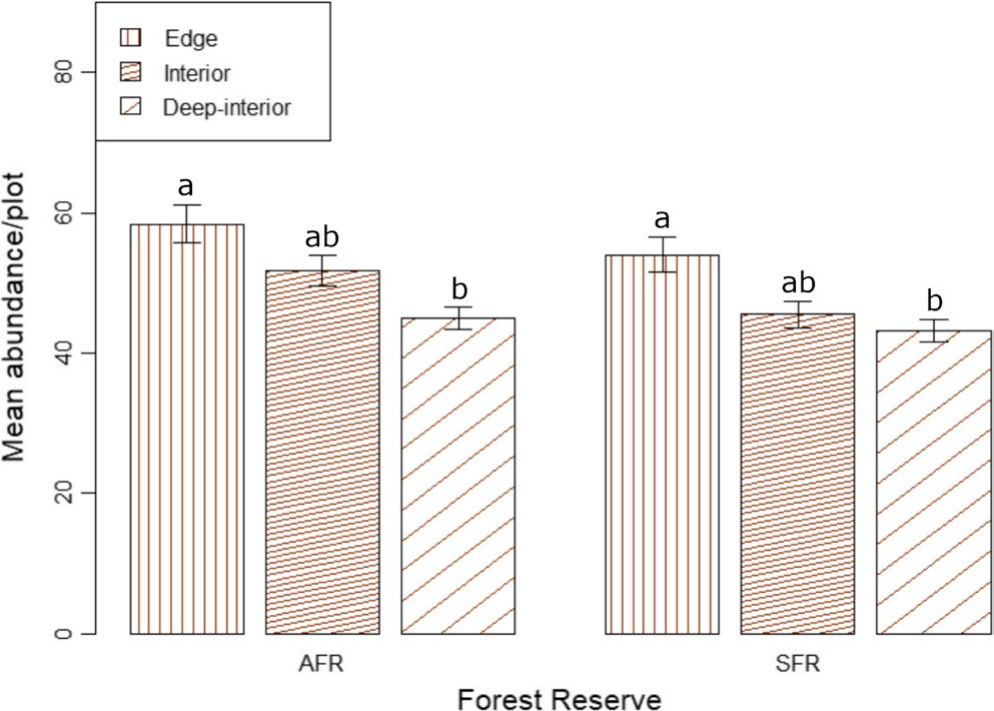
Mean liana abundance per plot within three forest sites in two moist semi‐deciduous forests in Ghana (AFR: Asenanyo Forest Reserve; SFR: Suhuma Forest Reserve). Within the same forest reserve, different letters indicate significantly different means among the forest sites as determined by Tukey test. Error bars show standard error of means

The contribution of the five most abundant liana species to the total liana abundance in the forest sites of AFR were as follows: edge – 54%, interior – 55.1% and deep‐interior – 59.8% (Table 1; Appendix S1). In the case of SFR, the five most abundant liana species contributed 53.9, 53.1 and 37.5% of the total liana stems in edge, interior and deep‐interior sites, respectively. Liana abundance differed significantly between edge and deep‐interior sites of the AFR (*p* = .009) and SFR (*p* = .010) (Figure 2). Nonetheless, there were no significant differences in liana abundance between edge and interior sites (AFR: *p* = .382; SFR: *p* = .276), and interior and deep‐interior sites (AFR: *p* = .154; SFR: *p* = .926) in the two forests. In both forest reserves, there was no significant effect of sampling site on liana abundance (AFR: *F* = .091, *p* = .964; SFR: *F* = 2.16, *p* = .128).

MEI in AFR ranged from −1 to 0.92 (Table 1). More species experienced positive MEI on their abundance than those that had negative MEI on their abundance. *Caesalpinia cucullata* and *Combretum acutum* were the only species that experienced very strong MEI in AFR. *Paullinia pinnata* was the only species with strong MEI on its abundance. The rest of the species recorded moderate, weak and very weak MEI on their abundance. On the contrary, *A*. *pentagona* had no MEI on its abundance. In SFR, the MEI on liana species abundance ranged from −0.43 to 0.45 (Table 1). The majority of the liana species experienced positive MEI on their abundance. Nevertheless, there was no MEI on the abundance of *Strophanthus sarmentosus* in SFR. There was moderate MEI on the abundance of *Manniophyton fulvum* and *Neuropeltis prevosteoides*, while the MEI on the abundance of the remaining species was either weak or very weak.

### 3.2 Network metrics

We observed 179 interactions between 40 liana species and 38 tree species in edge site of AFR. A total of 123 and 119 interactions were recorded in interior (involving 34 liana species and 28 tree species) and deep‐interior (between 31 liana species and 35 tree species), respectively. On the part of SFR, 44 liana species interacted with 63 tree species in edge site and produced a total of 202 interactions. In interior site, 44 liana species interacted with 46 tree species, resulting in 173 interactions. We recorded an interaction involving 42 liana species and 46 tree species in deep‐interior site, giving rise to 175 interactions.

Connectance of the three networks was significantly lower than that of the null models (Table 2). The specialisation asymmetric values of the networks in AFR were close to zero, indicating weak asymmetry. The specialisation asymmetry value of interior site network in AFR was consistent with that of the null model; those of the other networks were significantly higher than randomised expectations. The networks in SFR did not only show weak asymmetry, but they also did not differ significantly from that expected by chance. The degree of specialisation differed considerably among the species in the three forest sites within AFR. The majority of the liana species (edge: 60%, interior: 57.1%, deep‐interior: 66.7%) had significantly higher degree of specialisation than that of the respective null models in AFR (Appendix S2). Correspondingly, the degree of specialisation varied widely among the species in the forest sites of SFR. Most of the species in edge (59.1%), interior (55.6%) and deep‐interior (63.6%) sites of SFR were significantly more specialised than expected by chance. In AFR, the proportion of tree species with higher specialisation than their null models (edge: 42.1%, interior: 42.9%, deep‐interior: 37.1%) was lower than the proportion of tree species that did not show higher specialisation than expected by chance (Appendix S3). A similar trend was recorded in SFR (edge: 39.7%, interior: 39.1%, deep‐interior: 45.7%). Generally, higher proportions of lianas than tree species showed higher specialisation than the null models.

**TABLE 2:** Patterns of network properties of liana‐tree interactions among three forest sites of two moist semi‐deciduous forests in Ghana

Network metric	Asenanyo Forest Reserve			Suhuma Forest Reserve		
	Observed	Null model	*p*‐value	Observed	Null model	*p*‐value
Edge						
Connectance	0.13	0.16	.001	0.06	0.10	.001
Specialisation asymmetry	−0.12	−0.07	.001	−0.02	−0.01	.125
H2	0.27	0.14	.001	0.24	0.14	.001
WNODF	11.63	17.82	.001	6.47	7.51	.108
Modularity	0.36	0.27	.001	0.44	0.36	.001
C‐score	9.15	9.56	.013	5.73	5.83	.281
Interior						
Connectance	0.14	0.18	.001	0.08	0.10	.001
Specialisation asymmetry	−0.02	−0.01	.213	0.01	0.02	.113
H2	0.32	0.16	.001	0.23	0.14	.001
WNODF	13.53	18.60	.001	7.98	8.82	.233
Modularity	0.41	0.28	.001	0.42	0.33	.001
C‐score	8.90	9.17	.113	7.04	7.25	.137
Deep‐interior						
Connectance	0.11	0.15	.001	0.08	0.13	.001
Specialisation asymmetry	−0.03	−0.01	.008	−0.05	0.04	.447
H2	0.33	0.15	.001	0.24	0.11	.001
WNODF	11.96	16.36	.008	5.27	5.87	.229
Modularity	0.41	0.25	.001	0.45	0.34	.001
C‐score	5.83	5.92	.433	9.16	9.25	.435

**FIGURE 3:**
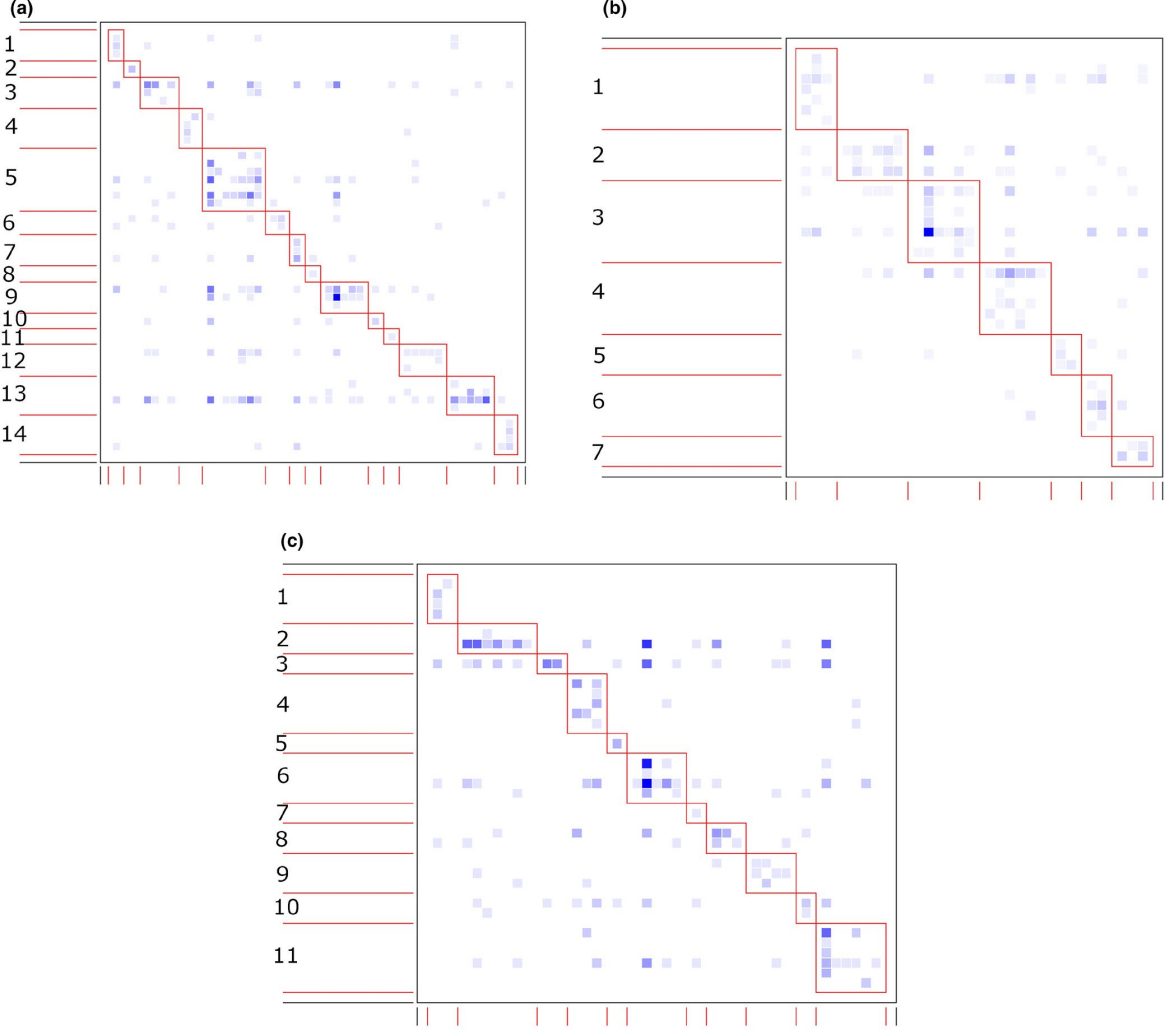
Network modules identified by DIRTLPAwb+ in three forest sites in Asenanyo Forest Reserve in Ghana [edge (a), interior (b), deep‐interior (c)]. The darker squares represent higher interaction frequency, while the light squares show lower frequency of interaction. The boxes show the modules of the networks, which are consecutively numbered. The species constituting the modules are found in Appendix S2

**FIGURE 4:**
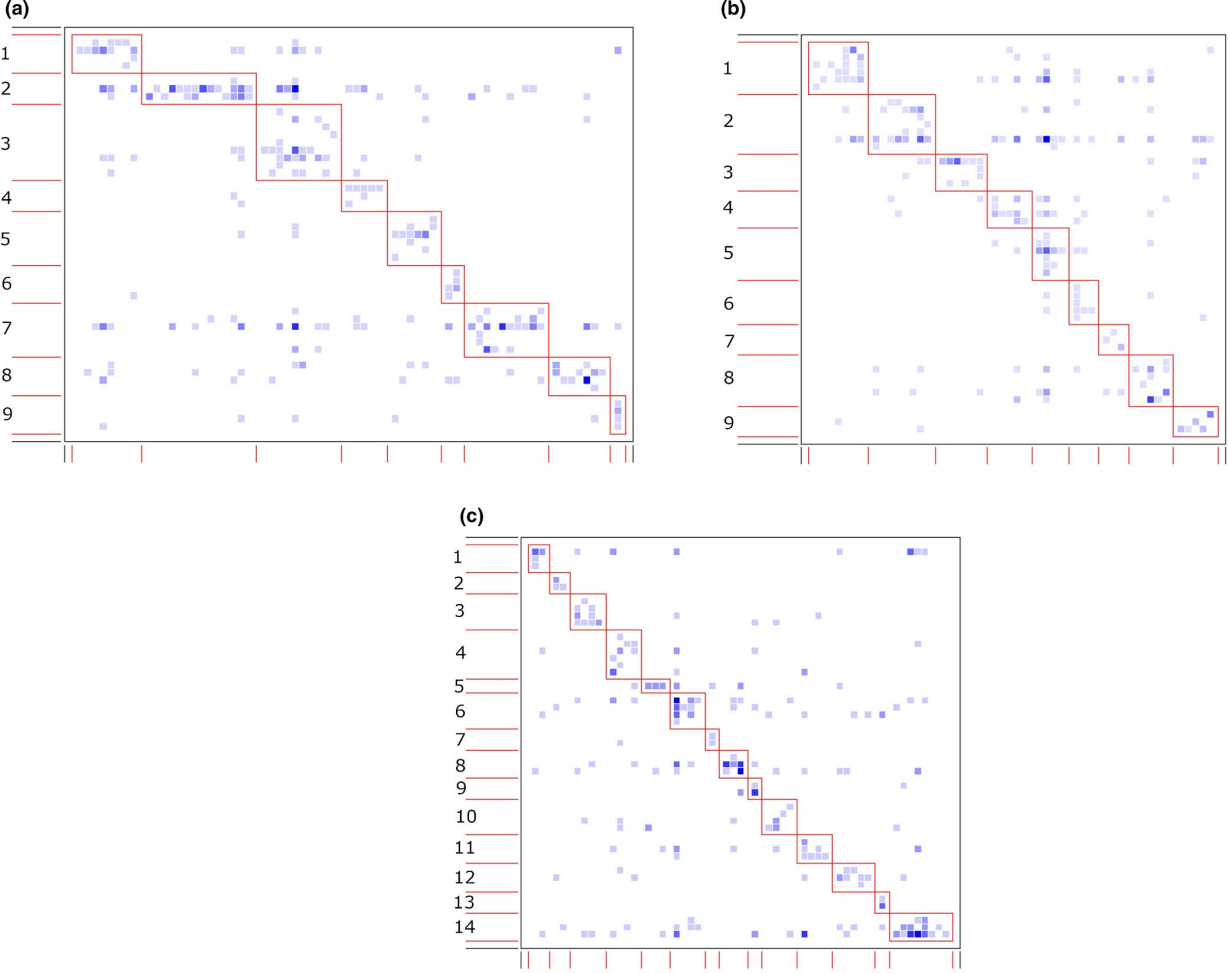
Network modules identified by DIRTLPAwb+ in three forest sites in Suhuma Forest Reserve in Ghana [edge (a), interior (b), deep‐interior (c)]. The darker squares represent higher interaction frequency, while the light squares show lower frequency of interaction. The boxes show the modules of the networks, which are consecutively numbered. The species constituting the modules are found in Appendix S2

In AFR, the observed nestedness metric values were significantly lower than the means of the null model in the three forest sites (Table 2). Likewise, the liana‐tree networks were less connected than the null models of the three networks. However, the three networks were more modular and specialised compared to the null networks. The significant modularity of the networks resulted in the formation of a number of modules in edge site (14 modules), which was more than the number of modules in deep‐interior (11 modules), which in turn, was more than that in interior site (7 modules) (Figure 3a–c; Appendix S4). The size of the modules varied greatly in the networks, ranging from 2 to 13 species in edge site, 5 to 13 species in interior site and 2 to 12 species in deep‐interior site. We did not observe significant differences in nestedness between the observed and null models in the three forest sites in SFR. Nevertheless, the three liana‐tree networks in the forest recorded significantly higher modularity and specialisation than expected by chance. The networks in deep‐interior forest site (deep‐interior: 14 modules) exhibited higher number of modules than the other sites (edge site: 9 modules, interior site: 9 modules) (Figure 4a–c; Appendix S4). Furthermore, the networks showed much variation in the size of the modules in the two forests (edge: 5–19 species, interior: 6–15 species, deep‐interior: 3–11 species).

### 3.3 Species topological roles in the networks

**FIGURE 5:**
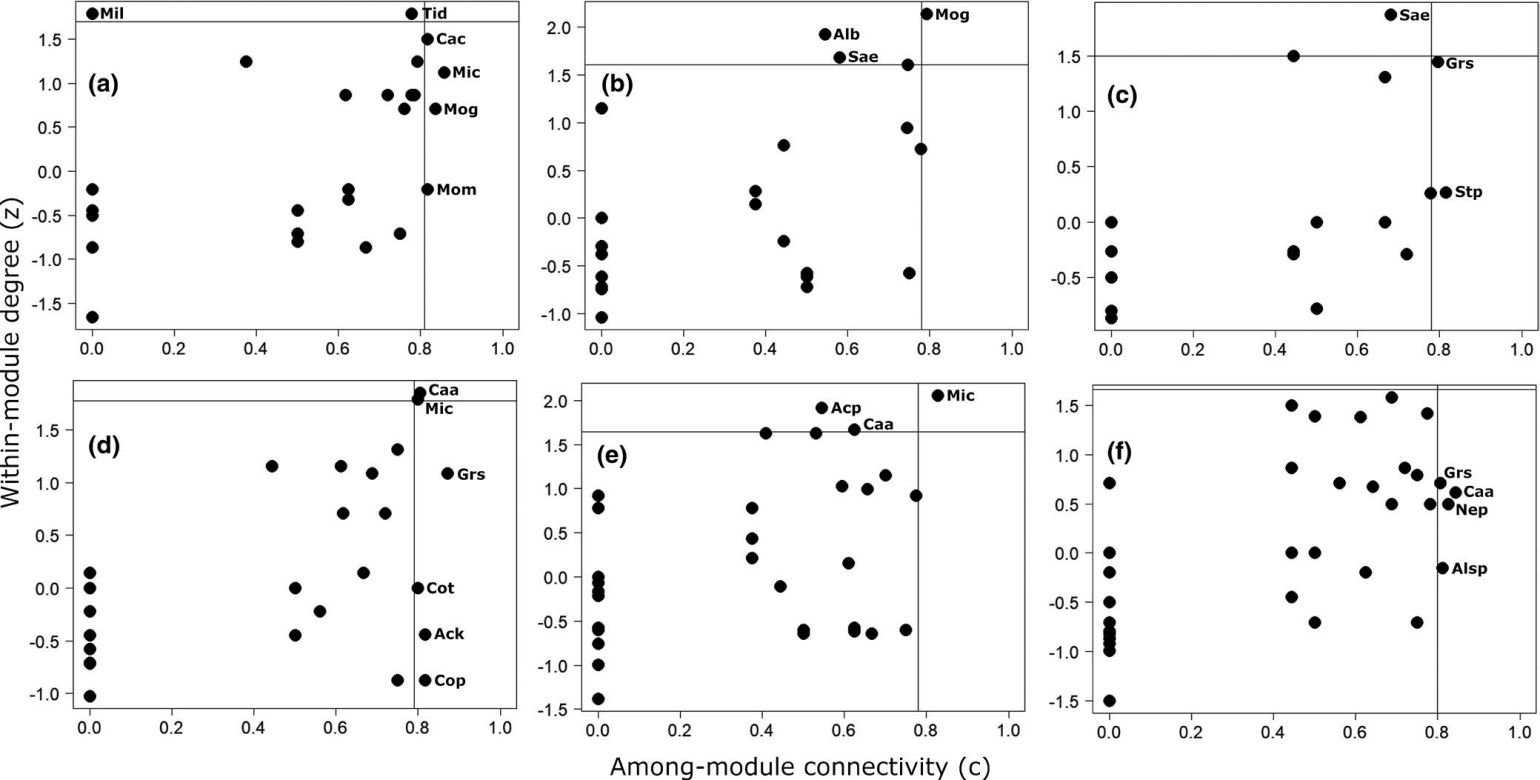
Module connectivity and interactions plots of the networks that show liana species roles within three sites in Asenanyo and Suhuma Forest Reserve (AFR and SFR, respectively) in Ghana [AFR edge (a), AFR interior (b), AFR deep‐interior (c), SFR edge (d), SFR interior (e), SFR deep‐interior (f)]. The threshold values of among‐module connectivity (*c*) and within‐module interaction (*z*) which were obtained from 95% quantiles from 100 null models are denoted by the vertical and horizontal lines. Species names are abbreviated to first two letters of the genus name and at least the first letter of the specific epithet (see Appendix S2 for full species names)

**FIGURE 6:**
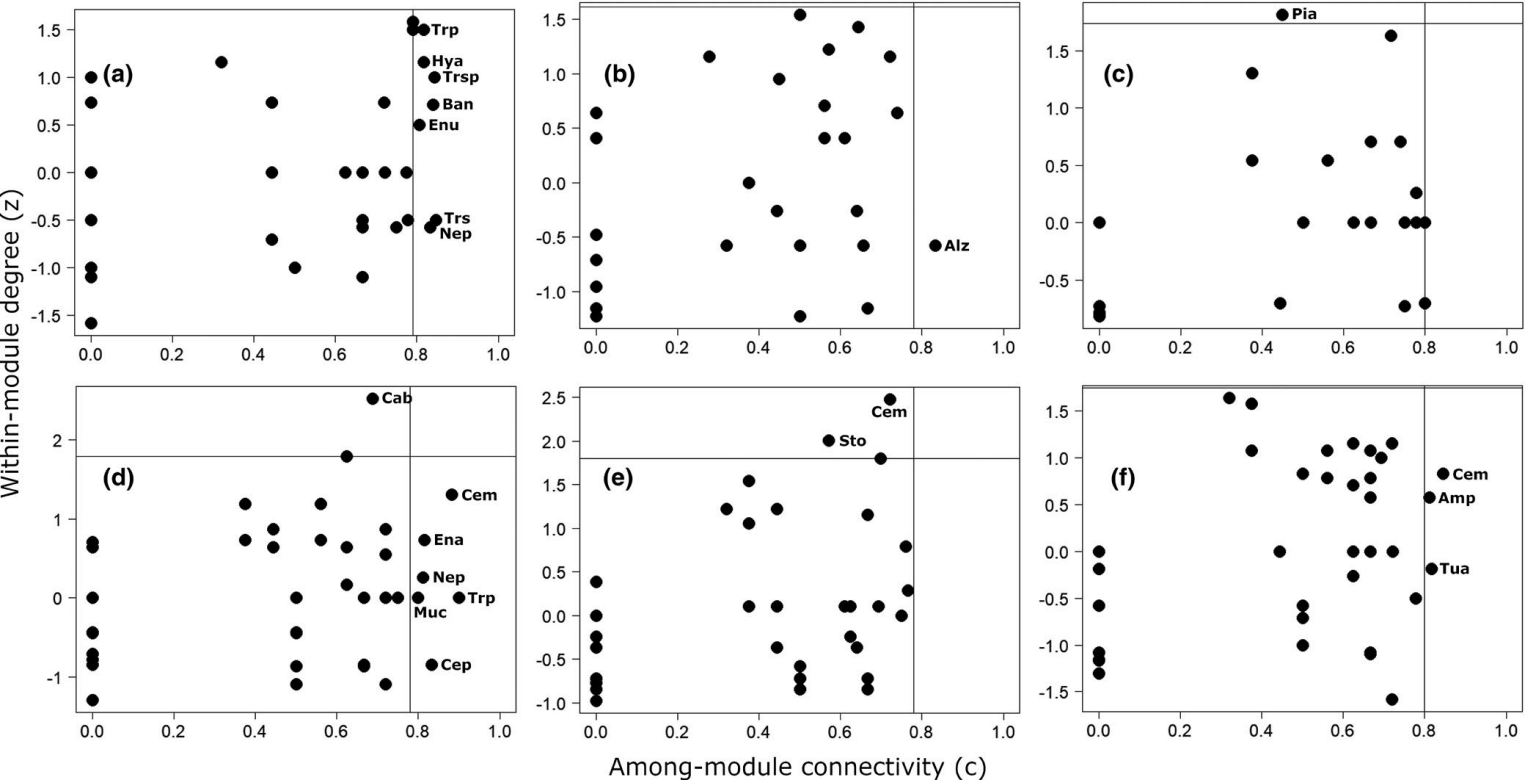
Module connectivity and interactions plots of the networks that show tree species roles within three sites in Asenanyo and Suhuma Forest Reserve (AFR and SFR, respectively) in Ghana [AFR edge (a), AFR interior (b), AFR deep‐interior (c), SFR edge (d), SFR interior (e), SFR deep‐interior (f)]. The threshold values of among‐module connectivity (*c*) and within‐module interaction (*z*) which were obtained from 95% quantiles from 100 null models are denoted by the vertical and horizontal lines. Species names are abbreviated to first two letters of the genus name and at least the first letter of the specific epithet (see Appendix S2 for full species names)

In AFR, liana species in edge site were mainly peripherals, with the exception of four species (*C*. *cucullata*, *M*. *chrysophylla*, *M*. *guineensis*, *Morinda morindoides*), which acted as connectors (Figure 5a). *Millettia lutens* and *Tiliacora dielsiana* were the only module hub species of lianas in edge site. Network hubs did not occur among lianas in edge site. The connector and module hub species constituted 15.4% of liana species in this site. In interior site, we had no liana connectors, but three module hubs (*A*. *barteri*, *S*. *elegans*, *M*. *chrysophylla*) and one network hub (*M*. *guineensis*) existed in this site, making up 11.8% of liana species in interior site (Figure 5b). The rest of the liana species served as peripherals in interior site. Within deep‐interior site, we recorded two liana connectors (*G*. *simplicifolia* and *S*. *preussii)* and one liana module hub (*S*. *elegans*) (Figure 5c), which together made up 10.3% of the liana species in the site. There was no network hub liana species in deep‐interior site. The rest of the liana species in deep‐interior site were peripherals. The majority of the tree species performed specialist role in edge site, but seven of the species (*C*. *mildbraedii*, *Hymenostegia afzelii*, *Trilepisium* sp., *Baphia nitida*, *Entandrophragma utile*, *Triplochiton scleroxylon*, *N*. *papaverifera*) were connectors (Figure 6a). These generalist species formed 25% of the tree species. Trees in interior site were mostly peripherals, with only one connector (*Albizia zygia*) and one module hub (*Berlinia confusa*) species (Figure 6b), but no network hub trees. These generalists were 5.7% of the total tree species in this site. There were three connectors (*C*. *mildbraedii*, *Amphimas pterocarpoides*, *Turraeanthus africanus*) and one module hub of tree species (*Homalium dewevrei*) in deep‐interior site, but there was no network hub species (Figure 6c). These tree species composed of 8.6% of the total number of species in deep‐interior site.

**FIGURE 7:**
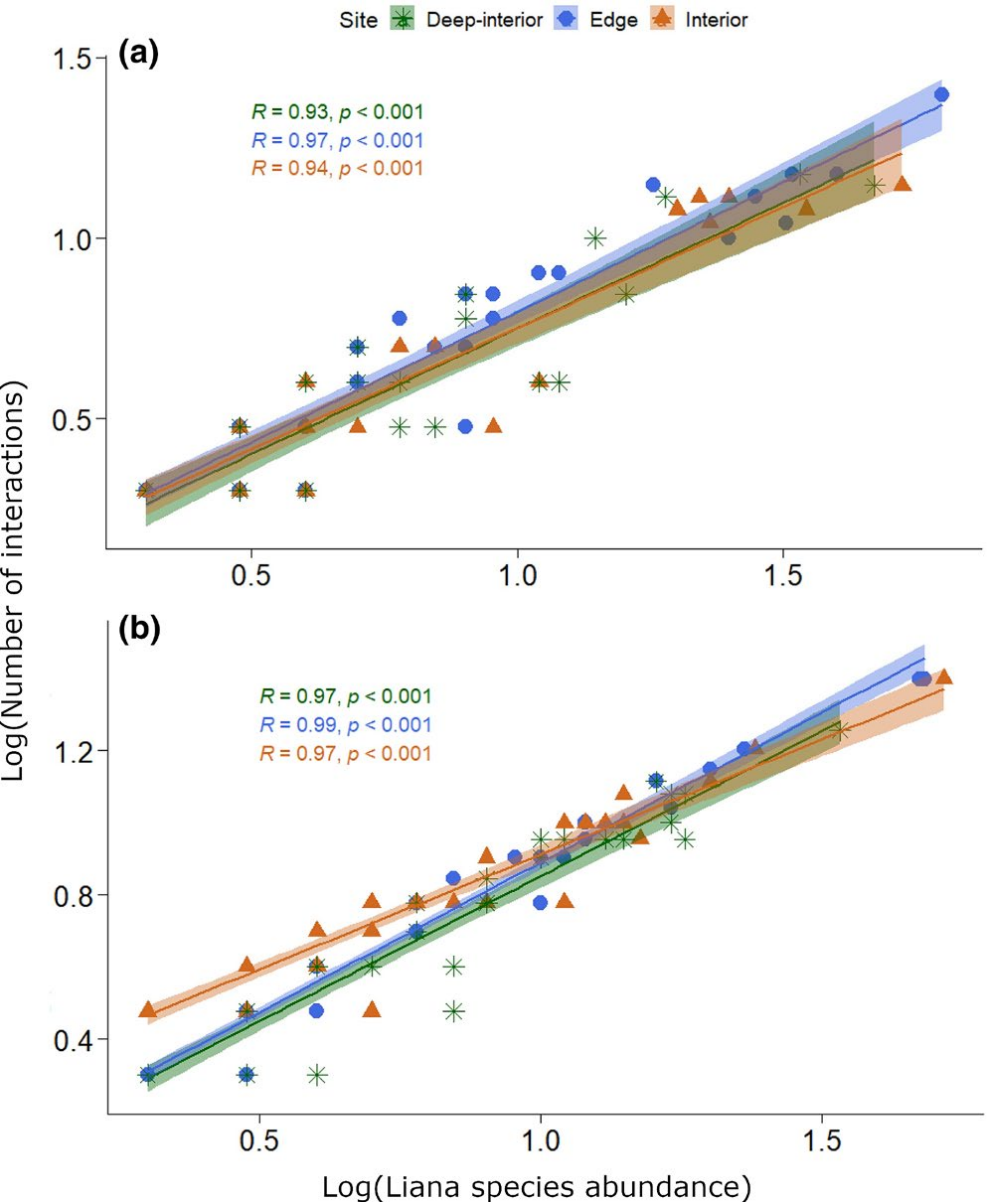
Relationships between liana species abundance and their number of interactions in edge, interior and deep‐interior sites of (a) Asenanyo Forest Reserve, and (b) Suhuma Forest Reserve

We recorded *G*. *simplicifolia*, *C*. *tarquense*, *A*. *kamerunensis* and *Combretum paniculatum* as connector liana species within edge site of SFR, while the majority of the liana species were peripherals (Figure 5d). *C*. *africanus* was a network hub in edge site. The above mentioned generalists constituted 11.4% of the total liana species. We did not record liana module hubs in edge site. In interior site of SFR, most of the liana species were peripherals. Generalist liana species were module hubs (*A*. *pentagona*, *C*. *africanus*, *N*. *acuminata*, *M*. *fulvum*) and network hub (*M*. *chrysophylla*), but with no connector species (Figure 5e). The above mentioned generalist species formed 12.2% of liana species in interior site. In deep‐interior site, lianas were mainly peripherals, except for *G*. *simplicifolia*, *C*. *africanus*, *Neuropeltis prevosteoides* and *Alafia* sp., that acted as connectors (Figure 5f). The above‐mentioned generalists formed about 9.8% of the total liana species. In deep‐interior site, module and network hubs were absent. Five tree species acted as connectors in edge site (*C*. *mildbraedii*, *Celtis philippensis*, *Entandrophragma angolense*, *N*. *papaverifera*, *Trichilia prieuriana*), while one tree species was identified as a module hub (*Calpocalyx brevibracteatus*) (Figure 6d). The rest of the tree species in edge site were peripherals. Network hubs of tree species were not recorded in edge site. Together, the connector and module hub species formed 9.5% of the total number of tree species in edge site. The majority of the tree species in interior site of SFR acted as peripherals. We did not identify connector tree species in this site, but a few module hub species occurred there (*Albizia adianthifolia*, *C*. *mildbraedii*, *Sterculia oblonga*) (Figure 6e). These generalist species formed 6.5% of the tree species. Network hubs of trees were not observed in interior site. Tree species in deep‐interior site were generally peripherals, except *C*. *mildbraedii* and *Ricinodendron heudelotii* (connectors), and *A*. *pterocarpoides* and *Guarea thompsonii* (module hubs) which formed 8.7% of the tree species (Figure 6f). We did not record tree network hubs in deep‐interior site. There were significant correlations between liana species abundance and their corresponding number of interactions in the edge, interior and deep‐interior sites of both AFR (Figure 7a) and SFR (Figure 7b).

### 3.4 Species co‐occurrence of lianas

The matrix in edge site of AFR showed positive co‐occurrence pattern, as the observed c‐score of the matrix in edge site was significantly lower than the mean of the null model (Table 2). In interior and deep‐interior sites of AFR, the c‐scores of the observed matrices were consistent with the simulated mean c‐scores, indicating random co‐occurrence. Similarly, all the matrices in the three sites in SFR, revealed random co‐occurrence pattern, since the observed c‐score values were not significantly different from those expected by chance.

## 4 DISCUSSION

### 4.1 Liana community structure

Our study showed contrasting edge effects on liana species diversity in the two moist semi‐deciduous forests. In AFR, edge appeared to have enhanced diversity, while an opposite trend occurred in SFR. The trend in AFR is consistent with previous studies that also recorded higher liana diversity at forest edges in relation to forest interiors (Addo‐Fordjour & Owusu‐Boadi, 2016; Laurance et al., 2001). As mentioned previously, SFR was heavily disturbed by conventional logging prior to edge disturbance. Consequently, further disturbance through edge creation caused a reduction in tree density at edge site (185 tree individuals/ha at edge compared to 242 tree individuals/ha at deep‐interior site) which may be related to edge‐induced mortality. Considering that limited availability of trees hampers liana species diversity (Addo‐Fordjour et al., 2012; Campbell et al., 2015), we attribute the lower liana diversity at the edge of SFR to the decreased tree density at the forest edge. It is important to note that edge effects on liana species diversity in our study occurred beyond interior sites of the two forests, penetrating over 200 m into the deep‐interior sites. This trend is at variance with what was reported in some rainforests in Ghana in which edge effects on liana species diversity were not detected up to 100 m (Ofosu‐Bamfo et al., 2019). The contrasting edge types of the two studies might be responsible for the different edge penetration distance with respect to liana diversity. Whereas the edge sites in Ofosu‐Bamfo et al. (2019) were narrow and adjacent to similar forest vegetation, our edge sites were wide and adjacent to farmlands. The effects of edge disturbance on liana species diversity at edge site of SFR occurred via a reduction in species evenness as indicated by our results. The forest edge appears to have influenced species evenness by exerting differential effects on the abundance of different species in edge site. Thus, in edge site of SFR, changes in species abundance mediated edge effects on liana species diversity. The observed species diversity variation between edge and interior sites of the two forests may possibly relate with edge‐related changes in variables such as microclimate, and tree density and mortality that often characterise forest edges (Ofosu‐Bamfo et al., 2019; Wekesa et al., 2019).

Our study revealed pronounced edge effects on liana abundance at the community level in the two forest reserves. This pattern is supported by previous studies which showed that edge effects enhanced liana abundance in their respective forests (Addo‐Fordjour & Owusu‐Boadi, 2016; Campbell et al., 2018; Laurance et al., 2001). Our results showed that in both forest reserves, edge effects on liana abundance penetrated 200 m from the edge, and this is in keeping with Laurance (1991) who reported that edge effects on the abundance of disturbance‐adapted plants such as lianas can penetrate 200 m into forest interior. Forest edges are often characterised by increased levels of light and desiccation or dryness, which can promote liana increase (Campbell et al., 2018). Given that there was a sharp contrast between our forest edges and the surrounding matrix, we expected the above mentioned conditions to be more pervasive in the edge sites. Thus, increased levels of light and dry conditions at the forest edge may be associated with the positive response of liana abundance to edge disturbance in the two forests. At the species level, many liana species showed diverse responses to edge disturbance in the two forests as evidenced by the wide range of MEI values. A similar finding was reported in two rainforests in Ghana (Ofosu‐Bamfo et al., 2019). Generally, many liana species in tropical forests are classified as light demanders (de Campos Franci et al., 2016), and therefore are expected to thrive in open areas. Consistent with this, some of our liana species showed high positive MEI, which indicates that they tended to prefer edge sites probably due to their light‐demanding nature. Nevertheless, the possession of negative MEI by other species shows that although optimal light and dry conditions might have characterised the forest edges, not all the lianas thrived as edge‐adapted species. From these results, there appears to be a spectrum of light tolerance physiology in the liana species at the edges of the forests. Such variation in the response of liana species abundance to edge disturbance could shape the network structure in ways that permit species to evolve or drive the evolution of the liana communities (Bascompte et al., 2003; Cantor et al., 2017; Gómez et al., 2011; Hansen, 2003).

### 4.2 Liana‐tree network structure

We found anti‐nested and modular structure in the three liana‐tree interaction networks in AFR. This trend has also been reported by Addo‐Fordjour and Afram (2021), and Addo‐Fordjour et al. (2021), and to some extent by Magrach et al. (2016) whose liana‐tree networks showed anti‐nested structure (see supplementary data in Magrach et al., 2016). Nevertheless, our results contrast with Sfair et al. (2010), who recorded nested structure in three distinct vegetation formations in Brazil, and also differs from the networks of Sfair et al. (2015), which did not show modularity. In SFR, all the three networks were not nested but modular. Though the two nestedness patterns shown by the networks in the AFR and SFR refer to non‐nested structure, that of the former depicts non‐random assembly of species, whereas the latter indicates random assembly of species. We argue that a clear distinction should be made between the two types of non‐nestedness in network studies so that the distribution pattern of each of them would be fully understood. The absence of nestedness in AFR and SFR may be due to differences in liana species ability to colonise host trees and/or the use of defence strategies of hosts to avoid lianas (Addo‐Fordjour et al., 2016; Genini et al., 2012). As a recap, a nested structure is formed when there are interactions involving generalists and generalists, and specialists and generalists, but no interaction of specialists and specialists (Landi et al., 2018). Staniczenko et al. (2013) showed that for a nested quantitative network, interactions of generalist–generalist species are strongest, followed by those of generalist–specialist species, with no specialist–specialist interactions (or when present with much weaker interactions). Thus, for a nested structure to occur in a quantitative network like ours, there should be a good number of specialist and generalist species undergoing interactions. However, in our networks, we observed only a few generalists of lianas and trees that interacted, but with many specialist species interacting among themselves. This situation increased the likelihood of specialist–specialist interactions at the expense of generalist–generalist and generalist–specialist interactions, resulting in the absence of nested structure in the various networks. A similar trend was observed in mycorrhizal networks (Jacquemyn et al., 2015). The specialist–specialist interactions in our networks may account for the non‐asymmetry and weak asymmetry of the networks. This finding shows that our networks tended to be more symmetric in their interactions, a trend which causes absence of nestedness and significant modularity in ecological networks (Guimarães et al., 2007). Overall, the findings on liana‐tree network structure reported in the current and previous studies show that there is no universal pattern in the structure of liana‐tree interactions. The patterns obtained may be dependent on the network complexity, and species traits and abundance, which are known to influence the organisation of liana‐tree interactions (Sfair et al., 2010, 2018). The existence of high modular structure in the various networks may increase their stability and robustness by limiting diffusion of perturbations through the networks (Thébault & Fontaine, 2010). This may explain why the patterns of network structure in edge site were consistent with those in interior and deep‐interior sites, irrespective of disturbance at edge site. The presence of modular structure in our networks may help conserve the networks of species interaction, which in turn, may lead to the conservation and maintenance of ecosystem functioning. The modular structure can enhance the stability of the liana communities in the various sites, and increase their robustness to perturbations (Olesen et al., 2007). When lianas are connected to many trees within a community, they tend to make the trees susceptible to fall, because during natural or artificial disturbance, lianas connected to falling trees may pull down other trees connected to them. However, module formation in networks may limit the pulling effects of lianas on trees to only affected modules, thereby conserving species in the other modules.

Liana‐tree interaction tends to be antagonistic, as lianas act as structural parasites of trees and compete intensely with trees for resources (Sfair et al., 2015, 2018). Species of antagonistic networks often evolve high specialisation in order to survive the antagonism of the interactions (Maliet et al., 2020). Our results revealed strong species and network specialisation in the forest sites, which implies the existence of strong liana‐host specificity across the various networks in the two forest. Network specialisation and host specificity have been reported to cause non‐nestedness and modularity in networks (Cordeiro et al., 2020; Dallas & Cornelius, 2015; Maliet et al., 2020; Wardhaugh et al., 2015). Given this information, the non‐nested and modular structure observed in our networks may be driven by specialisation of the networks and host specificity of the liana species. The specialisation in the liana‐tree networks may be related to co‐evolution in lineages of lianas and trees in the networks (Sfair et al., 2015). The possibility of co‐evolution of lianas and trees in our networks is supported by Ponisio and M'Gonigle (2017), who showed that ecological communities that co‐evolve become more anti‐nested and modular over time. Montoya et al. (2015) found out that functional group diversity increases with modularity in complex networks, and that functional groups form modules in communities. In this regard, the presence of high number of modules per network in the forest sites may reflect the existence of different liana functional groups that interact with tree communities in the forests. Such networks with high level of modularity may possess increased resistance to disturbance (Olesen et al., 2007; Saunders & Rader, 2019). Differences in colonisation rates in fish parasites were found as a cause of anti‐nested structure in such networks (Poulin & Guégan, 2000). In each of the networks, different liana species showed varying degree of specialisation, while others exhibited generalisation. This phenomenon suggests that the rates of colonisation differ markedly among the species, with highly specialised species having lower rate of colonisation, while species with low specialisation, or generalisation exhibit higher colonisation rate. In this regard, like the parasite‐fish networks (Poulin & Guégan, 2000), the anti‐nested structure in our networks could have partly been occasioned by variation in colonisation rates of the liana species. Generally, our study adds to the number of studies that have demonstrated the existence of non‐nestedness and modularity in liana‐tree networks (e.g. Addo‐Fordjour & Afram, 2021; Addo‐Fordjour et al., 2021; Magrach et al., 2016).

### 4.3 Species role in the networks

The finding of this study showed that lianas and trees were predominantly specialists (i.e. peripherals), irrespective of edge disturbance or edge effects, indicating possible robustness of species roles to disturbance. A similar pattern was recorded in some moist and dry semi‐deciduous forests in Ghana (Addo‐Fordjour & Afram, 2021; Addo‐Fordjour et al., 2021). As stated earlier, the specialist role or specialisation of the species might have resulted from antagonism between lianas and trees in the networks (Maliet et al., 2020). The role of some of the liana and tree species was consistent in the forest sites, while other species roles changed from one site to another. This phenomenon indicates that edge effects probably caused a switch in the role of some of the species among the forest sites, while the role of other species remained unchanged. Liana species abundance related positively with their number of interactions in all the sites in the two forests. This relationship shows that the abundant liana species in the forests tend to be generalists, whereas less abundant or rare species were specialists (Vázquez et al., 2005). The above‐mentioned relationship suggests that the switch from specialist to generalist and vice versa, and from one form of generalist to another by some species in AFR and SFR are perhaps related to changes in species abundance and distribution following edge disturbance (Addo‐Fordjour & Afram, 2021). For example, the abundance, and distribution (i.e. number of tree species hosting the lianas) of *M*. *chrysophylla* and *C*. *cucullata* decreased from edge site to deep‐interior site in AFR. These shifts resulted in changes in their topological role as connectors in edge site to module hub in interior site and peripheral in deep‐interior site for *M*. *chrysophylla*, and peripheral in interior and deep‐interior site for *C*. *cucullata*. A similar trend occurred in some of the tree species in the forests. Some of the species identified as structural important species (i.e. connectors, module hubs, network hubs) in our study were also reported as species that possessed structural importance in a moist semi‐deciduous forest in Ghana (Addo‐Fordjour & Afram, 2021; Addo‐Fordjour et al., 2021). These plants which include two liana species (*G*. *simplicifolia*, *C*. *africanus*) and three tree species (*T*. *scleroxylon*, *N*. *papaverifera*, *C*. *mildbraedii*) may have unique functional roles that support the functioning of the forests.

### 4.4 Species co‐occurrence of lianas

Generally, lianas were assembled randomly on their hosts in most of the forest sites, suggesting that chance events rather than edge disturbance, determined liana distribution on trees. Thus, we argue that the liana communities might have been assembled on trees by stochastic processes including host characteristics. Our finding is consistent with that reported in a semi‐deciduous forest in Brazil (Zulqarnain et al., 2016). Contrary to the above, liana species in edge site of AFR showed positive species co‐occurrence on their hosts. Since this network was organised into modules, the positive co‐occurrence trend could have existed within the modules. Thus, in the modules, liana species resorted to positive or facilitative interactions (McGarvey & Veech, 2018), that might have arisen deterministically. At forest edges, there is usually an elevated level of light coupled with dry conditions, and trellis availability, all of which can work together to enhance liana proliferation (Campbell et al., 2018). It appears that as these resources are increased at edge, lianas tend to share rather than compete for them, resulting in their positive co‐occurrence on the host trees. The liana species aggregation on trees could have also arisen by facilitation, where increasing liana abundance at edge site would cause new liana individuals to use already climbing stems to climb trees (Pérez‐Salicrup et al., 2001).

### 4.5 Implications for forest management and conservation

This study presents findings on the response of liana communities and the structure of liana‐tree interaction networks to edge disturbance. Our findings highlight that the severity of edge effects on liana species diversity may be influenced by land‐use history prior to edge disturbance. This shows that edge effects on liana species diversity may not be universal but site‐specific, depending on historical events of the forest. As this information is an exception to the general understanding that forest edges enhance liana diversity, it expands our knowledge about liana species diversity response to forest edge, which potentially, could contribute towards the development of a more inclusive theory on forest edge. Our study also shows that fragmentation of already disturbed sites may hamper liana species diversity due to edge effects. The presence of antinested structure in the networks of our forest sites could have arisen from strong selection for liana‐host specificity (Dickie et al., 2017; Sfair et al., 2010), given that most of the liana and tree species were specialists. The specificity among the liana and tree species may reflect in the formation of modules in the forest sites. With this development, future disturbance in the sites may be localised to specific modules, thus resulting in the conservation of other species in the networks (Thébault & Fontaine, 2010). Forest disturbance involving felling of specific tree species in a module can cause the loss of lianas that show high specificity for the tree species, since such lianas may not find other nearby trees suitable to climb. The affected lianas may fall unto the ground and remain hostless. This may reduce the chances of the lianas surviving, given that lianas growing on the ground are more susceptible to mortality than those growing on trees (Addo‐Fordjour et al., 2021). Therefore, forest managers can utilise the knowledge of host specificity of lianas and the existence of modules within networks to artificially regenerate affected trees and liana species in forests so as to restore the modules. Overall, our findings expand our understanding of edge effects on liana communities and liana‐tree interactions, and add to existing literature with respect to edge effects on plant communities and plant–plant interactions. Thus, the findings of this study provide valuable information which may be useful in developing a comprehensive edge theory that could be employed in managing and conserving forests.

## 5 CONCLUSION

The findings of the study revealed considerable edge effects on liana diversity and abundance in the two moist semi‐deciduous forests. The response of liana diversity to edge effects was positive in AFR, while a negative response was recorded in SFR. Despite the enhanced abundance in edge site of the two forests, the patterns of liana‐tree network structure of edge site were similar to those in interior and deep‐interior sites. The networks in AFR showed anti‐nested structure, while the networks in SFR revealed a nestedness pattern which was consistent with the null models. All the networks in the two forests were less connected, but modular and specialised. Lianas were mostly randomly distributed on host trees in all the forest sites except edge site in SFR. Topologically, the majority of liana and tree species were peripherals (i.e. specialist), but a few species tended to be generalists, acting as connectors, module hubs and network hubs. The role of most of the species did not change from one site to another, even though the topological role of a few species changed from one site to another. Overall, our study shows that liana community structure was more susceptible to forest edge than liana‐tree network structure. The findings of our study corroborate previous studies, and also present unique findings related to liana‐tree network structure. Our findings which enhance our understanding of liana‐tree interactions, have conservation implications relating to stability and robustness of the networks. Finally, the findings of the present study can potentially contribute to the development of a comprehensive theory on edge effects.

## CONFLICT OF INTEREST

The authors declare no conflict of interest.

## AUTHOR CONTRIBUTIONS

**Bismark Ofosu‐Bamfo:** Formal analysis (supporting); Investigation (lead); Methodology (supporting); Writing – original draft (equal); Writing – review & editing (supporting). **Patrick Addo‐Fordjour:** Conceptualization (lead); Formal analysis (lead); Methodology (lead); Supervision (equal); Writing – original draft (equal); Writing – review & editing (lead). **Ebenezer J.D. Belford:** Conceptualization (supporting); Methodology (supporting); Writing – original draft (supporting); Writing – review & editing (supporting).

## Supporting information

Appendix S1–S4

## Data Availability

The data associated with this study is available at dryad (https://doi.org/10.5061/dryad.crjdfn35t).
