# Supplementary material for: Edge disturbance shapes liana diversity and abundance but not liana‐tree interaction network patterns in moist semi‐deciduous forests, Ghana
Source: Ecol Evol. 2022 Feb 20;12(2):e8585. doi: 10.1002/ece3.8585 (PMC8859495; doi:10.1002/ece3.8585)
Supplement: Supplementary file 1 — Appendix S1–S4 [file ECE3-12-e8585-s001.docx]

**Appendix S1** The five most abundant liana species in three forest sites of two moist semi-deciduous forests in Ghana

| **Species** | **Edge** | **Interior** | **Deep-interior** |
| --- | --- | --- | --- |
| **Asenanyo Forest Reserve** |  |  |  |
| *Millettia chrysophylla* | 18.0 | 17.9 | 19.2 |
| *Salacia elegans* | 9.6 | 10.4 | 8.9 |
| *Griffonia simplicifolia* | 9.4 | 8.9 | 13.9 |
| *Alafia barteri* | 8.6 | 8.0 | 11.4 |
| *Motandra guineensis* | 8.4 | 9.9 | - |
| *Strophanthus preussii* | - | - | 6.4 |
| **Suhuma Forest Reserve** |  |  |  |
| *Millettia chrysophylla* | 19.0 | 23.1 | 9.8 |
| *Griffonia simplicifolia* | 14.6 | 9.1 | 7.2 |
| *Alafia barteri* | 4.6 | - | 6.4 |
| *Motandra guineensis* | 9.0 | 7.1 | - |
| *Calycobolus* *africanus* | 5.8 | 6.9 | 6.6 |
| *Acacia pentagona* | - | 6.9 | 7.5 |

**APPENDIX S2** Standardised specialisation index (*d’*) for liana species within liana-tree networks in three forest sites of two moist semi-deciduous forests, Ghana. Values with an asterisk are significantly higher than expected by chance. Species abbreviation (Sp. abb.) are shown in the table to facilitate reference to Figure 5 and Appendix 4.

| **Liana species** | **Sp. abb.** | **Asenanyo Forest Reserve** | | | **Suhuma Forest Reserve** | | |
| --- | --- | --- | --- | --- | --- | --- | --- |
|  |  | Edge | Interior | Deep-interior | Edge | Interior | Deep-interior |
| *Acacia kamerunensis* | Ack | 0.41* | - | 0.64* | 0.30 | 0.45* | 0.39* |
| *Acacia pentagona* | Acp | 0.47* | 0.34* | 0.54* | 0.55* | 0.65* | 0.32 |
| *Acridocarpus smeathmannii* | Acs | 0.66* | - | - | - | - | - |
| *Afrobrunnichia erecta* | Afe | 0.63* | 0.19* | 0.53* | - | - | 0.22 |
| *Agelaea obliqua* | Ago | - | 0.40* | - | - | - | 0.17 |
| *Agelaea trifolia* | Agt | 0.27 | 0.60* | 1.00* | 0.80* | 0.48* | - |
| *Alafia barteri* | Alb | 0.18 | 0.28 | 0.29 | 0.54* | 0.38* | 0.42* |
| *Alafia* sp. | Alsp | 0.39* | 0.30 | 0.30 | 0.43* | 0.13 | 0.17 |
| *Baphia capparidifolia* | Bac | - | - | - | 0.51* | - | - |
| *Caesalpinia cucullata* | Cac | 0.27 | 0.56* | 0.31 | - | 0.31 | 0.24 |
| *Calycobolus africanus* | Caa | 0.16 | 0.22 | 0.56* | 0.25 | 0.13 | 0.21 |
| *Calycobolus heudelotii* | Cah | 0.36* | - | - | - | 1.00* | 0.69* |
| *Castanola paradoxa* | Cap | - | - | - | 0.43* | - | - |
| *Chlamydocarya macrocarpa* | Chm | - | 0.54* | - | - | - | - |
| *Cissus adenocaulis* | Cia | 0.12 | 0.67* | 0.58* | 0.23 | 0.19 | 0.00 |
| *Cissus silvestris* Tchoume | Cis | - | 0.72* | - | - | - | - |
| *Clerodendrum* sp. | Clsp | - | - | - | 0.30 | - | - |
| *Cnestis ferruginea* | Cnf | - | - | - | - | 0.31 | - |
| *Combretum acutum* | Coac | - | 0.53* | - | - | 0.44* | - |
| *Combretum comosum* | Coc | 0.31 | 0.13 | 0.44* | - | - | 0.71* |
| *Combretum fuscum* | Cof | - | - | - | - | - | 0.41* |
| *Combretum micranthum* | Comi | 0.66* | - | - | - | - | - |
| *Combretum mucronatum* | Comu | - | - | - | 0.39* | 0.03 | 0.42* |
| *Combretum oyemense* | Coo | 0.33* | 0.08 | 0.45* | - | - | - |
| *Combretum paniculatum* | Cop | 0.34* | 0.54* | - | 0.48* | 0.24 | 0.43* |
| *Combretum racemosum* | Cor | - | - | - | 0.14 | 0.87* | 0.44* |
| *Combretum sordidum* | Cos | - | - | - | 0.55* | - | - |
| *Combretum* sp. | Cosp | - | - | - | - | 0.31 | - |
| *Combretum tarquense* | Cot | - | - | - | 0.19 | 0.62* | 0.22 |
| *Connarus africanus* | Coaf | - | - | - | - | 0.64* | - |
| *Dalbergia hostilis* | Dah | 0.43* | 0.13 | - | 0.25 | 1.00* | 0.67* |
| *Dalbergia oblongifolia* | Dao | - | - | 0.09 | - | - | - |
| *Dalbergiella welwitschii* | Daw | 0.32* | 0.30 | 0.52* | 0.35 | 0.13 | 0.49* |
| *Dichapetalum dewevrei* | Did | - | - | - | 0.60* | - | - |
| *Dichapetalum pallidum* | Dip | - | 0.34* | 0.59* | 0.53* | - | - |
| *Ficus* sp. | Fisp | - | - | - | - | - | 0.42* |
| *Gongronema latifolium* | Gol | - | - | - | - | 0.44* | - |
| *Grewia hookeriana* | Grh | 0.66* | 0.24 | 0.81* | - | - | - |
| *Grewia malacocarpa* | Grm | - | - | - | 0.45* | 0.52* | 0.56* |
| *Griffonia simplicifolia* | Grs | 0.11 | 0.28 | 0.24 | 0.26 | 0.17 | 0.40* |
| *Hippocratea myriantha* | Him | - | - | - | - | - | 0.45* |
| *Hugonia planchonii* | Hup | - | - | - | - | 0.63* | - |
| *Hugonia rufipilis* | Hur | - | - | - | 0.53* | - | - |
| *Illigera pentaphylla* | Ilp | - | - | - | 1.00* | - | 0.45* |
| *Landolphia dulcis* | Lad | 0.22 | - | 0.24 | 0.43* | 0.00 | 0.49* |
| *Landolphia hirsuta* | Lah | - | - | - | 0.08 | 0.34 | 0.26 |
| *Landolphia owariensis* | Lao | - | - | - | 0.80* | 0.81* | - |
| *Leptoderris cyclocarpa* | Lec | - | - | - | 0.09 | - | - |
| *Leptoderris micrantha* | Lemic | 0.62* | 0.54* | 0.54* | 0.68* | 0.08 | 0.68* |
| *Leptoderris miegei* | Lemie | 0.11 | - | - | 0.43* | - | 0.47* |
| *Leptoderris sassandrensis* | Les | - | - | - | 0.30 | 0.38* | - |
| *Leucomphalos libericus* | Lel | - | - | - | 0.53* | - | - |
| *Manniophyton fulvum* | Maf | - | 0.82* | - | 0.62* | 0.38* | 0.36 |
| *Mezoneuron benthamianum* | Meb | - | - | - | 0.26 | - | - |
| *Millettia chrysophylla* | Mic | 0.25 | 0.28 | 0.35* | 0.35 | 0.16 | 0.34 |
| *Millettia lucens* | Mil | 0.75* | - | 0.59* | - | - | - |
| *Morinda morindoides* | Mom | 0.21 | 0.59* | - | - | - | - |
| *Motandra guineensis* | Mog | 0.20 | 0.20 | 0.26 | 0.45* | 0.22 | 0.32 |
| *Mussaenda tristigmatica* | Mut | 0.66* | - | - | - | - | - |
| *Neuropeltis acuminata* | Nea | 0.26 | 0.45* | 0.44* | 0.47* | 0.28 | 0.33 |
| *Neuropeltis prevosteoides* | Nep | - | - | 0.43* | 0.29 | 0.63* | 0.27 |
| *Oncinotis nitida* | Onn | - | - | - | - | - | 0.35 |
| *Parquetina nigrescens* | Pan | - | - | - | - | 0.00 | - |
| *Paullinia pinnata* | Pap | 0.52* | 0.43* | 0.85* | - | - | - |
| *Phyllanthus* sp. | Pisp | - | - | - | - | - | 0.41* |
| *Piper guineense* | Pig | 0.29 | - | - | - | 0.46* | 0.80* |
| *Salacia cerasifera* | Sac | - | - | - | - | 0.37* | - |
| *Salacia debilis* | Sad | 0.92* | - | - | 0.53* | 0.33 | 0.48* |
| *Salacia elegans* | Sae | 0.24 | 0.31 | 0.23 | 0.23 | 0.18 | 0.38* |
| *Salacia lateritia* | Sala | 0.67* | 0.16 | - | - | - | - |
| *Salacia leptoclada* | Sale | - | - | - | - | 0.69* | - |
| *Salacia macrantha* | Sam | - | - | - | - | 0.63* | - |
| *Salacia preussii* | Sap | - | 0.45* | - | - | - | - |
| *Salacia staudtiana* | Sas | - | - | - | - | 0.81* | - |
| *Salacighia letestuana* | Salet | - | - | - | 0.69* | - | 0.79* |
| *Simirestis staudtii* | Sis | - | - | - | - | - | 0.63* |
| *Strophanthus hirsutus* | Sthir | 0.29 | - | - | 0.61* | - | - |
| *Strophanthus hispidus* | Sthis | 0.73* | - | - | - | - | - |
| *Strophanthus preussii* | Stp | 0.37* | 0.19 | 0.16 | - | - | - |
| *Strophanthus sarmentosus* | Sts | - | - | - | 0.30 | 0.70* | 0.35 |
| *Strychnos campicola* | Stc | 0.51* | 0.42* | 0.59* | - | - | - |
| *Strychnos longicaudata* | Stl | - | - | - | 0.34 | 0.40* | 0.50* |
| *Strychnos malacoclados* | Stm | - | - | - | - | 0.10 | 0.48* |
| *Tetracera affinis* | Tea | 0.52* | 0.40* | 0.42* | - | - | - |
| *Tiliacora dielsiana* | Tid | 0.38* | 0.26 | 0.44* | 0.38* | 0.49* | 0.49* |
| *Triclisia patens* Oliv. | Trp | 0.83* | 0.53* | 0.29 | - | - | 1.00* |

**APPENDIX S3** Standardised specialisation index (*d’*) for tree species within liana-tree networks in three forest sites of two moist semi-deciduous forests, Ghana. Values with an asterisk are significantly higher than expected by chance. Species abbreviation (Sp. abb.) are shown in the table to facilitate reference to Figure 6 and Appendix 4.

| **Tree species** | **Sp. abb.** | **Asenanyo Forest Reserve** | | | **Suhuma Forest Reserve** | | |
| --- | --- | --- | --- | --- | --- | --- | --- |
|  |  | Edge | Interior | Deep-interior | Edge | Interior | Deep-interior |
| *Aidia genipiflora* | Aig | 0.16 | - | - | 1.00* | - | - |
| *Albizia adianthifolia* | Ala | 0.39* | 0.20 | 0.17 | 0.23 | 0.37* | - |
| *Albizia ferruginea* | Alf | - | - | - | 0.63* | 0.22 | - |
| *Albizia zygia* | Alz | 0.00 | 0.36* | 0.18 | 0.52* | - | 0.29 |
| *Alstonia boonei* | Alb | - | - | 0.13 | 0.49* | - | - |
| *Amphimas pterocarpoides* | Amp | - | 0.55* | - | 0.30 | 1.00* | 0.39* |
| *Annickia polycarpa* | Anp | - | - | - | 0.42* | - | - |
| *Anopyxis klaineana* | Ank | - | - | 0.07 | - | - | - |
| *Anthocleista* sp. | Ansp | - | - | - | 0.28 | - | - |
| *Anthonotha fragrans* | Anf | - | - | - | - | - | 0.41* |
| *Anthonotha macrophylla* | Anma | - | - | - | 0.00 | - | - |
| *Antiaris toxicaria* | Ant | 0.11 | - | - | 0.37* | - | 0.15 |
| *Antrocaryon micraster* | Anmi | - | - | - | - | - | 0.37* |
| *Baphia nitida* | Ban | 0.29 | 0.10 | 0.39* | 0.34 | 0.15 | 0.28 |
| *Baphia pubescens* | Bap | 0.16 | - | - | 0.25 | 0.69* | 0.12 |
| *Berlinia confusa* | Bec | 0.12 | 0.33* | 0.09 | - | 0.54* | - |
| *Berlinia occidentalis* | Beo | - | - | - | - | - | 0.41* |
| *Blighia sapida* | Bls | - | - | - | 0.15 | 0.39* | 0.27 |
| *Bombax buonopozense* | Bob | - | - | - | 0.34 | - | - |
| *Buchholzia coriacea* | Buc | - | - | - | - | 0.32 | - |
| *Bussea occidentalis* | Buo | - | - | - | 0.26 | 0.82* | - |
| *Calpocalyx* *brevibracteatus* | Cab | - | - | - | 0.27 | 0.18 | 0.33 |
| *Carapa procera* | Cap | - | - | 0.42* | 0.28 | 0.65* | 0.31 |
| *Canarium schweinfurtii* | Cas | - | - | - | 0.21 | - | - |
| *Cedrela odorata* | Ceo | 0.33* | 0.19 | 0.24 | 0.00 | - | - |
| *Ceiba pentandra* | Cep | - | 0.43* | 0.65* | 0.17 | 0.14 | - |
| *Celtis adolfi-friderici* | Cea | - | 0.50* | - | 0.24 | - | - |
| *Celtis mildbraedii* | Cep | 0.16 | 0.27 | 0.29 | 0.18 | 0.14 | 0.17 |
| *Celtis philippensis* | Cem | - | - | - | 0.12 | - | - |
| *Celtis zenkeri* | Cez | - | - | - | 0.11 | - | - |
| *Chrysophyllum perpulchrum* | Chp | - | - | 0.42* | - | - | - |
| *Chrysophyllum subnudum* | Chs | 0.48* | 0.38* | - | - | - | - |
| *Cola chlamydantha* | Coc | - | - | - | 0.34 | 0.46* | 0.12 |
| *Cola gigantea* | Cog | - | 0.46* | 0.09 | - | 0.37* | 0.52* |
| *Cola nitida* | Con | 0.14 | - | - | - | - | - |
| *Corynanthe pachyceras* | Cop | - | - | 0.66* | - | 0.08 | 0.23 |
| *Cuviera nigrescens* | Cun | - | 0.34* | - | - | - | - |
| *Dacryodes klaineana* | Dak | - | - | - | 0.80* | - | 0.41* |
| *Daniela* sp. | Dasp | - | - | - | - | - | 0.31 |
| *Desplatsia* sp. | Desp | 0.73* | - | - | - | 0.39* | - |
| *Dialium aubrevillei* | Dia | - | - | - | 0.37* | 0.20 | 0.45* |
| *Diospyros gabunensis* | Dig | - | - | - | - | 0.32 | 0.71* |
| *Diospyros kamerunensis* | Dik | - | - | - | - | - | 0.60* |
| *Diospyros sanza-minika* | Dis | - | - | - | 0.28 | - | 0.41* |
| *Discoglypremna* sp. | Discsp | - | 0.17 | - | - | - | - |
| *Distemonanthus* sp. | Distsp | - | - | - | - | 0.65* | - |
| *Drypetes gilgiana* | Drg | - | 0.11 | - | - | - | - |
| *Drypetes principum* | Drp | - | - | - | 0.21 | - | - |
| *Elaeis guineensis* Jacq. | Elg | - | - | 0.25 | - | - | - |
| *Entandrophragma angolense* | Ena | 0.94* | 0.22 | 0.38* | 0.16 | - | 0.33 |
| *Entandrophragma cylindricum* | Enc | 0.23 | - | - | - | - | - |
| *Entandrophragma utile* | Enu | 0.21 | 0.89* | 0.25 | 0.27 | - | - |
| *Ficus exasperata* | Fie | - | - | - | 0.34 | 0.18 | - |
| *Ficus sur* | Fis | 0.51* | - | - | - | - | - |
| *Funtumia africana* | Fua | 0.09 | - | 0.37* | - | - | 0.64* |
| *Funtumia elastica* | Fue | - | - | - | 0.60* | - | - |
| *Guarea thompsonii* | Gut | - | 0.30 | - | - | - | 0.31 |
| *Guibourtia ehie* | Gue | - | 0.11 | - | - | - | - |
| *Hannoa klaineana* | Hak | - | - | - | 0.37* | - | - |
| *Hevea brasiliensis* | Heb | - | - | - | 0.23 | - | - |
| *Hexalobus crispiflorus* | Hec | - | 0.27 | - | - | - | - |
| *Homalium dewevrei* | Hod | - | - | - | - | - | 0.40* |
| *Hymenostegia afzelii* | Hya | 0.22 | 0.26 | 0.77* | 0.28 | - | 0.23 |
| *Irvingia gabonensis* | Irg | 0.45* | - | 0.28 | - | - | 0.32 |
| *Lannea welwitschii* | Law | - | 0.59* | - | 0.38* | - | 1.00* |
| *Lonchocarpus sericeus* | Los | 0.37* | - | 0.30 | - | - | - |
| *Lovoa trichilioides* | Lot | - | - | - | - | 0.24 | - |
| *Macaranga barteri* | Mab | - | - | - | 0.00 | - | - |
| *Mammea africana* | Mama | - | - | 0.55* | - | - | - |
| *Margaritaria discoidea* | Mad | - | - | - | 0.64* | - | - |
| *Microdesmis puberula* | Mip | 0.22 | - | - | 0.19 | 0.18 | - |
| *Milicia excelsa* | Mie | - | - | - | - | - | 0.40* |
| *Musanga cecropioides* | Muc | - | - | - | 0.42* | 0.38* | - |
| *Massularia acuminata* | Masa | - | - | - | - | 0.25 | - |
| *Myrianthus arboreus* | Mya | - | - | - | 0.43* | 0.56* | 0.54* |
| *Myrianthus libericus* | Myl | - | - | - | 0.20 | 0.36 | 0.40* |
| *Napoleonaea vogelii* | Nav | 0.31* | - | - | - | 0.18 | - |
| *Nauclea diderrichii* | Nad | - | - | 0.25 | - | - | - |
| *Nesogordonia papaverifera* | Nep | 0.26 | 0.00 | 0.16 | 0.22 | 0.30 | 0.25 |
| *Newbouldia laevis* | Nep | 0.56* | - | 0.00 | - | - | - |
| *Omphalocarpum ahia* | Oma | - | - | - | - | 0.39 | - |
| *Pachystela msolo* | Pam | - | - | - | - | - | 0.54* |
| *Pachystela brevipes* | Pab | - | - | - | - | 0.00 | - |
| *Parinari excelsa* | Pae | - | - | - | 0.32 | - | - |
| *Parkia bicolor* | Pab | - | - | - | - | - | 0.37* |
| *Pentaclethra* sp. | Pesp | - | - | - | - | - | 0.00 |
| *Petersianthus macrocarpus* | Pem | 0.53* | - | - |  |  |  |
| *Piptadeniastrum africanum* | Pia | 0.87* | 0.19 | 0.45* | 0.46* | 0.41* | 0.23 |
| *Pterygota macrocarpa* | Ptm | - | - | - | 0.58* | 0.29 | 0.20 |
| *Pycnanthus angolensis* | Pya | 0.72* | 0.39* | 0.49* | - | 0.35 | 0.23 |
| *Rauvolfia vomitoria* | Rav | 0.63* | - | 0.21 | 0.59* | 0.37* | - |
| *Ricinodendron heudelotii* | Rih | 0.54* | 0.47* | - | 0.22 | 0.26 | 0.28 |
| *Rinorea* sp. | Risp | - | - | - | 0.86* | - | - |
| *Scottellia klaineana* | Sck | - | - | 0.18 | 0.33 | 1.00* | 0.69* |
| *Sterculia oblonga* | Sto | - | - | 0.49* | 0.37* | 0.29 | - |
| *Sterculia rhinopetala* | Str | - | - | - | 0.16 | 0.05 | - |
| *Sterculia tragacantha* | Stt | 0.13 | - | - | 0.23 | - | - |
| *Strombosia pustulata* | Stp | 0.18 | 0.22 | 0.23 | 0.79* | 0.09 | 0.28 |
| *Synsepalum aubrevillei* | Sya | - | - | - | 0.00 | - | - |
| *Terminalia ivorensis* | Tei | - | - | - | 0.58* | - | - |
| *Terminalia superba* | Tes | - | 0.17 | - | - | - | 0.43* |
| *Tetrapleura tetraptera* | Tet | 0.10 | - | - | - | - | - |
| *Treculia africana* | Tra | - | - | - | - | 0.07 | - |
| *Trichilia monadelpha* | Trm | 0.12 | 0.11 | 0.29 | 0.41* | 0.35 | 0.31 |
| *Trichilia prieuriana* | Trp | 0.23 | - | 0.00 | 0.11 | 0.20 | 0.17 |
| *Trichilia tessmannii* | Trt | - | - | - | - | 0.37* | - |
| *Tricalysia disco*lor | Trd | - | - | 1.00* | - | - | - |
| *Trilepisium* sp. | Trsp | 0.18 | - | - | 0.12 | 0.45* | 0.00 |
| *Triplochiton scleroxylon* | Trs | 0.10 | - | 0.22 | 0.55* | - | - |
| *Turraeanthus africanus* | Tua | - | - | - | - | 0.34 | 0.40* |
| *Uapaca corbisieri* | Uac | 0.90* | - | - | - | - | - |
| *Zanthoxylum gilletii* | Zag | 0.13 | - | 0.25 | 0.38* | - | - |
| *Zanthoxylum parvifoliolum* | Zap | - | - | - | 0.78* | - | - |

**APPENDIX S4** Liana and tree species forming modules in the networks of the forest sites in the two moist semi-deciduous forests in Ghana. Liana species are indicated in **bold** text, while tree species are not bolded. The species are represented by codes, which are made up of first two letters of the genus name and at least the first letter of the specific epithet (see Appendices 1 and 2 for the abbreviations and their corresponding full names).

| **Module #** | **Asenanyo Forest Reserve** | | | **Suhuma Forest Reserve** | | |
| --- | --- | --- | --- | --- | --- | --- |
|  | Edge | Interior | Deep-interior | Edge | Interior | Deep-interior |
| Module 1 | **Cia, Daw, Pig,** Trs | **Cop, Grh, Mog, Stc, Tea, Chm, Cac,** Ala, Cep, Discsp | **Agt, Mil, Nep, Afe,** Cap, Trd | **Alsp, Caa, Cap, Lao,** Ala, Amp, Buo, Cep, En.an, Ptm, Sck, Coc | **Coaf, Cop, Cot, Daw, Grs, Sas,** Cap, Dia, Distsp, Fie, Ptm, Str, Ich | **Mog, Daw, Lad,** Dia, Trp |
| Module 2 | **Sad,** Pia | **Agt, Alb, Daw, Grs,** Ena, Gut, Gue, Hec, Tes, Rih | **Ack, Mic,** Alz, Alb, Chp, Nep, Nel, Sck, Trp | **Lah, Mic, Mog,** Alb, Anma, Bap, Bls, Cea, Cep, Cez, Dis, Drp, Mab, Mad, Nep, Trp, Trt | **Ack, Alb, Cnf, Hup, Les, Mic, Tid**  Cab, Cog, Lot, Mip, Nav, Rav, Sto, Stp | **Afe, Sas,**  Anmi, Cop |
| Module 3 | **Mog, Nea, Trp,** Hya, Nep, Nel, Aig | **Caa, Coac, Coo, Dah, Mic, Nea, Tid,** Amp, Cem, Drg, Nep, Pia, Stp | **Alb,** Fua, Ena | **Clsp, Coso, Did, Ilp, Les, Nep, Sae, Sts, Nea,**  Ant, Ban, Cap, Cea, Cem, Fie, Hak, Heb, Zag, Aig | **Acp, Grm, Pig, Salep,** Bls, Buo, Desp, Mya, Oma, Tua | **Dah, Fisp, Maf, Tid,** Anf, Dak, Dik, Stp |
| Module 4 | **Afe, Grh, Mil, Mut,** Ena, Uac | **Sae, Sala, Sap, Stp, Cia, Trp,** Ban, Be.co, Ce.od, Ch.su, Py.an, Tr.mo | **Acp, Cac, Coo, Lemic, Tea,** Cep, Los, Pia | **Maf, Stl, Tid,** Anp, Mip, Myl, Tei, Trm | **Alsp, Gol, Mog, Sas,** Cep, Cap, Trm, Trp, Trsp | **Cof, Cop, Cot, Sts, Phsp, Cac,** Ban, Dig, Mie, Rih |
| Module 5 | **Cah, Coo, Cop, Grs, Lad, Sae, Mom,** Cem, Pya, Stt, Trp, Trs, En.ut, | **Ago, Alsp, Maf,** Cea, Etu | **Pap,** Ala | **Agt, Daw, Grm, Hur, Lemie, Salet,** Alf, Bob, Fue, Hya, Rav, Rid | **Lad, Lemic, Nea, Pan, Sae, Stm,** Ban, Cem, Dig, Tra | **Alb**  Beo, Dis, Ptm |
| Module 6 | **Acp, Stp,** Pem, Rav | **Acp, Afe, Coc, Pap. Dip,** Hya, Law | **Alsp, Caa, Grs, Mog,** Bec, Ceo, Cog, Irg, Ra.vo | **Dip, Lel, Sthir, Lad,** Sck, Zap | **Cac, Comu, Cosp, Lah, Sad,** Ala, Buc, Mua | **Caa, Lah, Nea, Cia,** Cem, Pab, Pia, Coc |
| Module 7 | **Ack, Agt, Cac,** Ban | **Lemic, Mom,** Alz, Cog, Cui | **Cia,** Ank | **Cia, Cop, Grs, Lec, Lemic, Nea,** Cas, Dak, Law, Mya, Pae, Str, Stt, Sya, Rih, Enu | **Cah, Lao, Nep,** Pya, Sck, Trt | **Lemic, Stm,** Fua |
| Module 8 | **Sthir,** Bap |  | **Nea, Tid,** Enu, Mama, Sto | **Ack, Acp, Alb, Sad,** Alz, Ansp, Cap, Dia, Muc, Pia, Sto | **Agt, Caa, Cia, Dah, Maf, Stl,** Alf, Amp, Nep, Pia, Coc | **Ilp, Sae, Nep,** Cog, Ena, Hod |
| Module 9 | **Alb, Alsp, Lemie,** Ant, Ceo, Enc, Mip, Zag |  | **Caa, Daw, Stc,** Cop, Hya, Trm, Trs | **Comu, Cos, Cot, Dah,** Tr.sc | **Cosm, Sam, Sts,** Bap, Bec, Muc, Myl, Pab | **Onn, Stl,** Tua |
| Module 10 | **Coc,** Irg |  | **Stp, Dip,** Ban |  |  | **Cah, Trp, Comu, Grm,** Alz, Cab, Law, Dasp |
| Module 11 | **Comi,** Alz |  | **Coc, Dao, Lad, Sae, Trp, Grh,** Cem, Elg, Nad, Pya, Stp, Zag |  |  | **Ack, Alsp, Lemie,** Amp, Mya, Myl, Pai |
| Module 12 | **Caa, Stc, Acs,**  Chs, Fua, Nav, Tet, Rih |  |  |  |  | **Sad, Cosm, Pig,**  Bap, Bls, Gut, Sck, Tes |
| Module 13 | **Dah, Lemic, Mic, Sthis,** Ala, Bec, Fis, Stp, Los |  |  |  |  | **Him, Coc,** Trm |
| Module 14 | **Pap, Sala, Tea, Tid**  Con, Desp |  |  |  |  | **Acp, Grs, Mic**  Ant, Cap, Hya, Irg, Nep, Pesp, Pya, Trsp |
